# Siglec-E retards atherosclerosis by inhibiting CD36-mediated foam cell formation

**DOI:** 10.1186/s12929-020-00698-z

**Published:** 2021-01-05

**Authors:** Yaw-Wen Hsu, Fu-Fei Hsu, Ming-Tsai Chiang, Dong-Lin Tsai, Fu-An Li, Takashi Angata, Paul R. Crocker, Lee-Young Chau

**Affiliations:** 1 Institute of Biomedical Sciences, Academia Sinica, No.128, Sec.II, Academy Road, Taipei, 115 Taiwan; 2 Institute of Biological Chemistry, Academia Sinica, Taipei, 115 Taiwan; 3 Division of Cell Signaling and Immunology, School of Life Sciences, University of Dundee, Dow Street, Dundee, DD1 5EH Scotland, UK

**Keywords:** Atherosclerosis, Sialic acid, Siglec-E, Macrophages, CD36, Low-density lipoprotein

## Abstract

**Background:**

The accumulation of lipid-laden macrophages, foam cells, within sub-endothelial intima is a key feature of early atherosclerosis. Siglec-E, a mouse orthologue of human Siglec-9, is a sialic acid binding lectin predominantly expressed on the surface of myeloid cells to transduce inhibitory signal via recruitment of SH2-domain containing protein tyrosine phosphatase SHP-1/2 upon binding to its sialoglycan ligands. Whether Siglec-E expression on macrophages impacts foam cell formation and atherosclerosis remains to be established.

**Methods:**

ApoE-deficient (apoE^−/−^) and apoE/Siglec-E-double deficient (apoE^−/−^/Siglec-E^−/−^) mice were placed on high fat diet for 3 months and their lipid profiles and severities of atherosclerosis were assessed. Modified low-density lipoprotein (LDL) uptake and foam cell formation in wild type (WT) and Siglec-E^−/−^- peritoneal macrophages were examined in vitro. Potential Siglec-E-interacting proteins were identified by proximity labeling in conjunction with proteomic analysis and confirmed by coimmunoprecipitation experiment. Impacts of Siglec-E expression and cell surface sialic acid status on oxidized LDL uptake and signaling involved were examined by biochemical assays.

**Results:**

Here we show that genetic deletion of Siglec-E accelerated atherosclerosis without affecting lipid profile in apoE^−/−^ mice. Siglec-E deficiency promotes foam cell formation by enhancing acetylated and oxidized LDL uptake without affecting cholesterol efflux in macrophages in vitro. By performing proximity labeling and proteomic analysis, we identified scavenger receptor CD36 as a cell surface protein interacting with Siglec-E. Further experiments performed in HEK293T cells transiently overexpressing Siglec-E and CD36 and peritoneal macrophages demonstrated that depletion of cell surface sialic acids by treatment with sialyltransferase inhibitor or sialidase did not affect interaction between Siglec-E and CD36 but retarded Siglec-E-mediated inhibition on oxidized LDL uptake. Subsequent experiments revealed that oxidized LDL induced transient Siglec-E tyrosine phosphorylation and recruitment of SHP-1 phosphatase in macrophages. VAV, a downstream effector implicated in CD36-mediated oxidized LDL uptake, was shown to interact with SHP-1 following oxidized LDL treatment. Moreover, oxidized LDL-induced VAV phosphorylation was substantially lower in WT macrophages comparing to Siglec-E^−/−^ counterparts.

**Conclusions:**

These data support the protective role of Siglec-E in atherosclerosis. Mechanistically, Siglec-E interacts with CD36 to suppress downstream VAV signaling involved in modified LDL uptake.

## Background

Atherosclerosis is the underlying cause of most cardiovascular diseases, including coronary heart disease, stroke, and peripheral vascular disease [1]. It is a complex pathological process initiated by the injury and activation of endothelium caused by hypercholesterolemia and other risk factors, followed by the accumulation and oxidative modification of low-density lipoproteins (LDLs) and the recruitment of circulating inflammatory cells, including monocytes and T lymphocytes, to the subendothelial intima [2–4]. The monocytes then differentiate into macrophages and uptake modified LDLs to become foam cells with a feature of intracellular cholesterol accumulation as lipid droplets, which is the hallmark of early lesion [2–4]. These foam cells in turn produce more inflammatory cytokines and growth factors to promote the migration of the quiescent vascular smooth muscle cells (VSMCs) in the medial layer to intima and activate VSMC proliferation and increased synthesis of extracellular matrix proteins, resulting in the thickening of intima and occlusion of the blood vessel [2–4]. At the advanced stage foam cells are unable to fully process the internalized cholesterol and eventually die, causing the deposition of cholesterol and the formation of lipid necrotic core in the arterial wall. This triggers further immune reaction to exacerbate the disease progression and provoke coronary rupture underlying heart attack [2–4]. It is apparent that macrophage-derived foam cells play a key role in the initiation and progression of atherosclerosis.

Siglecs are a family of sialic acid-binding immunoglobulin-like receptors predominantly expressed on cells of immune system to regulate their functions through recognizing their glycan ligands [5]. The cytoplasmic domains of most Siglecs contain the immunoreceptor tyrosine-based inhibitory motifs (ITIMS) which transduce negative signals via recruitment of SH2-domain containing protein tyrosine phosphatases, such as Src homology region 2 domain-containing phosphatase-1 (SHP-1) and SHP-2. Human Siglec-9 and its murine orthologue, Siglec-E, are mainly expressed on cells of the myeloid lineage, including neutrophils, monocytes/macrophages, and dendritic cells. Earlier studies have shown that overexpression of Siglec-9 enhances interleukin-10 (IL-10) but reduces tumor necrosis factor-α (TNF-α) production in macrophages [6]. It has been shown that ligands for Siglec-9 are up-regulated in human tumor samples and that tumor Siglec-9 ligands inhibit neutrophil activation in vitro, indicating that Siglec-9 is implicated in innate immune response to cancer [7]. Moreover, studies on Siglec-E-knockout mice have revealed that Siglec-E is a negative regulator of neutrophil recruitment to the inflammatory lung via suppressing CD11b β2-integrin-dependent signaling [8]. A recent study has also suggested that Siglec-E deficiency accelerates aging process in mice by modulating oxidative stress [9]. These findings highlight the importance of Siglec-9/E in the regulation of immune and inflammatory reactions in health and diseases. Nevertheless, the role of Siglec-9/E in vascular disease has not yet been explored. In an attempt to explore the role of Siglec-9/E in atherosclerosis, we generated apoE/Siglec-E double deficient (apoE^−/−^/SE^−/−^) mice to assess the effect of Siglec-E deletion on apoE^−/−^ mice, which is a mouse model developing hypercholesterolemia and atherosclerotic lesions similar to those in humans [10]. We show that Siglec-E deficiency facilitates vascular lesion formation in apoE^−/−^ mice. At the cellular level, Siglec-E inhibited foam cell formation via downregulating CD36-mediated modified LDL uptake in macrophages.

## Methods

### Plasmid constructs

N-terminal Flag tagged mouse CD36 plasmid (Flag-CD36) and N-terminal HA-tagged human CD36 plasmid (HA-CD36) were obtained from Sino Biological Inc. (Wayne, PA, USA). C-terminal Myc-Flag-tagged Siglec-9 plasmid (Flag-Siglec-9) was from OriGene (Rockville, MD, USA). The C-terminal HA-tagged wild type (WT) Siglec-E construct (HA-SE) was prepared by polymerase chain reaction (PCR) amplification of full length Siglec-E coding sequence from mouse spleen cDNAs using the following primers: 5′-AGAATTCATGCTGCTGTTGCTGCTGCT-3′ and 5′-CCTCGAGTCAAGCGTAATCTGGAACATCGTATGGGTATGGCCATGCGGTCCT-3′. The PCR product with HA-tag sequence fused to C-terminus of Siglec-E was digested with EcoRI and XhoI restriction enzymes and subcloned into pcDNA3 vector (Invitrogen, Waltham, MA, USA). The sialic acid binding defective mutant of Siglec-E (Siglec-E^R126D^) [8] with HA-tag was generated by site-directed mutagenesis using PCR.

### Animals

WT and Siglec-E^−/−^ (SE^−/−^) mice in C57BL/6 genetic background [8] were obtained from intercross between Siglec-E heterozygous (SE^±^) mice. To generate ApoE^−/−^SE^−/−^ mice, ApoE^−/−^ mice on C57BL/6 J background, which were originally obtained from Jackson laboratory (Bar Harbor, ME, USA) and maintained in our animal facility, were crossbred with SE^−/−^ mice to generate ApoE^±^/SE^±^ mice, which were backcrossed with apoE^−/−^ mice to produce ApoE^−/−^/SE^±^ mice. These mice were then intercrossed to generate ApoE^−/−^ mice bearing SE^+/+^, SE ^±^, and SE^−/−^ genotypes. To facilitate atherosclerotic lesion formation, ApoE^−/−^ and ApoE^−/−^SE^−/−^ mice at age of 8 to10-week old were placed on high-fat diet (HFD) (D12108Ci; Research Diets Inc. New Brunswick, NJ, USA) with 1.25% cholesterol and 40% fat kcal for 12 weeks. Mice were housed at ambient temperature of 21–23 °C and on a 12 h light–dark cycle with free access to food and water in specific pathogen-free condition. One week before sacrifice, blood samples were collected from animals. Serum high-density lipoprotein (HDL) and LDL separation was performed using HDL and LDL/VLDL cholesterol assay kit (ab65390; abcam, Cambridge, MA, USA). The levels of total cholesterol, HDL and LDL were measured by Infinity Cholesterol Reagent (401-100P; Sigma, St Louis, MO, USA). The serum triglyceride level was measured by dry chemistry analyzer Fuji Dri-Chem 4000i using FUJI DRI-CHEM SLIDE TG-PIII (Fujifilm Corporation, Tokyo, Japan). After animals were sacrificed, heart and aorta were removed from phosphate-buffered saline (PBS)-perfused animals, fixed in 4% paraformaldehyde.

### Pathological assessment

The thoracic-abdominal aortas were subjected to en face oil red dye (ORD)-staining and the luminal surface visualized by stereoscopic microscopy (SteREO Lumar V12; ZEISS, Hamburg, Germany). The images were analyzed by MetaMorph (7.7.5.0) software. Hearts were embedded in paraffin and sectioned at 5 um for histological examination and immunostaining. Total 40 serial sections from aortic sinus of each mouse were collected, and 5 sections sampled from every 10 consecutive sections were subjected to Trichrome staining with aniline blue (Sigma). To perform immunohistochemistry, sections were subjected to antigen retrieval in 10 mM Na-citrate pH 6.0 containing 0.05% Tween-20 at 98 °C for 20 min, followed by blocking with 5% donkey serum in PBS containing 0.2% Triton X-100 for 3 h at room temperature. Sections were then treated with 3% H_2_O_2_ to exhaust endogenous peroxidase activity, and incubated with control rat IgG (sc-2026, Santa Cruz, Dallas, TX, USA) or rat anti-F4/80 antibody (GTX26640, Genetex, Irvine, CA, USA) as indicated in PBS at 4 °C overnight. After three PBS washes, sections were incubated with horseradish peroxidase conjugated secondary antibody and the antigen–antibody complex was visualized by a 3′-diaminobenzidine liquid substrate system **(**Sigma**).** For quantitative analysis, the images were captured by Pannoramic 250 FLASH II Slide Scanner (3D-Histech®, Budapest, Hungary). The areas with F4/80^+^-macrophages, lesion sizes and percentages of necrotic core were quantified by MetaMorph (7.7.5.0) software.

### Peritoneal macrophage isolation

Peritoneal macrophages were isolated accordingly [11]. Briefly, mice were intraperitoneally injected with 1.5 ml of 3% thioglycollate (1.08191.0500, Merck, Kenilworth, NJ, USA). After 4 days, peritoneal macrophages were isolated by washing the peritoneal cavity with F12/Dulbecco's modified Eagle medium (F12/DMEM) containing 0.5% bovine serum albumin (BSA). Cells were then plated on culture plate or cover slips in medium containing 10% fetal bovine serum (FBS). After 4 h incubation, the nonadherent cells were removed and the adherent cells used as peritoneal macrophages. Unless specified, macrophages pooled from 3 mice of each genotype were used for comparative experiments.

### Real-time quantitative PCR

Total RNAs were extracted from peritoneal macrophages isolated from ApoE^−/−^ and ApoE^−/−^SE^−/−^ mice fed with HFD for 12 weeks using TRIzol reagent (Thermo Fisher Scientific, Waltham, MA, USA), followed by reverse-transcription using the Superscript III first strand cDNA synthesis kit (Thermo Fisher Scientific). Real-time PCR was performed using a LightCycler® FastStart DNA MasterPLUS SYBR Green I kit (Roche Applied Science, Upper Bavaria, Germany) on a LightCycler® Carousel-Based System (Roche Applied Science). The primer sequences used for PCR are listed as follows: TNFα (5′-AGACCCTCACACTCAGA-3′ and 5′-CCTTGTCCCTTGAAGAGAAC-3′), IL-6 (5′-GAGGATACCACTCCCAACAGACC-3′ and 5′-AAGTGCATCATCGTTGTTCATACA-3′), MCP-1 (5′-CTTCTGGGCCTGCTGTTCA-3′ and 5′-CCAGCCTACTCATTGGGATCA-3′), IL-10 (5′-GGTTGCCAAGCCTTATCGGA-3′ and 5′-ACCTGCTCCACTGCCTTG CT-3′), and IL-1β (5′-GATCCACACTCTCCAGCTGCA-3′ and 5′- CAACCAACAAGTGATATTCTCCATG-3′). GAPDH (5′-TGAAGGTCGGTGTGAACGGATTTG-3′ and 5′- TCTCGTGGTTCACACCCATCACAA-3′) was used as an internal control for normalization.

### Modified LDL preparation

To prepare acetylated LDL (acLDL), 1 mg of human LDL (360–10, Lee BioSolution, Maryland Heights, MO, USA) in 1 ml of 50% ice-cold saturated sodium acetate was incubated with 1.5 μl of acetic anhydride for 1 h at 4 °C with rotation [12]. The acLDL was then dialyzed against PBS containing 300 µM ethylenediaminetetraacetic acid (EDTA) and once more against PBS at 4 °C. To prepare 1,1′-dioctadecyl-3,3,3′,3′-tetramethylindocarbocyanine perchlorate (Dil)-labeled acLDL (Dil-acLDL), 1 ml of acLDL was incubated with 50 µl Dil-dye (3 mg/ml) in PBS at 37 °C for 18 h, followed by ultrafiltration using Microcon centrifugal filter (MRCF0R030, Merck Millipore, Darmstadt, Germany) against PBS to remove unbound Dil-dye. To prepare oxidized LDL (oxLDL), LDL (2 mg/ml) in PBS was incubated with 10 µM CuSO_4_ for 18 h at 37 °C [13]. Oxidation was stopped by adding 20 µM butylated hydroxytoluene and 300 µM EDTA. For Dil-oxLDL preparation, Dil-LDL was first prepared by incubation with Dil-dye as described above, followed by CuSO4 oxidation.

### Modified LDL binding and uptake

The binding and uptake of modified LDL were performed as described [14]. Macrophages plated on 48-well plates or glass coverslips were incubated with 10 µg/ml of Dil-acLDL or Dil oxLDL as indicated at 4 °C or 37 °C for indicated times. After 3 washes with Hank’s balanced saline solution (HBSS), cells were fixed and fluorescence were examined by confocal microscope and images analyzed by MetaMorph (7.7.5.0). To examine the effect of desialylation, macrophages were treated with 0.1 unit/ml of *Arthrobacter ureafaciens* sialidase (24229-74, Nacalai Tesque, Kyoto, Japan) in HBSS at 37 °C for 30 min prior to assay. In separate experiment, macrophages were pretreated with 10 µM of NSC87877, a potent inhibitor of SHP-1/2 phosphatase [15], for 30 min in culture before assay. To perform assay with HEK293T cells overexpressing HA-SE together with or without Flag-CD36, cells were incubated with 5 µg/ml of Dil-oxLDL at 37 °C for 1 h and examined by confocal microscopy as described above.

### Foam cell formation

Macrophages plated on 12-well plates were incubated with 50 µg/ml of acLDL or oxLDL for 24 h in culture. Cells were then fixed with 4% paraformaldehyde and stained with ORD [14].

### Cholesterol Efflux

Cholesterol efflux was determined using the cholesterol efflux fluorometric Assay Kit (Cat. K582100; BioVision, Milpitas, CA, USA). Briefly, macrophages were plated on 96-well plates (1X10^5^ /well) and labeled with fluorescently-labeled cholesterol analogue for 1 h. After equilibration in culture for 16 h, cells were incubated without (negative control) or with 10 mM methyl-β cyclodextrin (positive control) or 50 μg/ml human HDL(MBS173147, MyBioSource, San Diego, CA, USA) as indicated in phenol red-free, serum-free RPMI medium for 6 h. The culture medium and cell lysate were separately collected and the fluorescence intensity (RFU) was determined using a microplate reader (SpectraMax Gemini EM Microplate Reader, Molecular Devices, San Jose, CA, USA). The percentage of cholesterol efflux = [RFU of medium/(RFU of cell lysate + RFU of medium)] × 100.

### SILAC (Stable isotope labeling using amino acids in cell culture) labeling of Raw264.7 cells

Murine Raw264.7 cell line originally obtained from American Type Culture Collection (ATCC, Gaithersburg, MD, USA) was maintained in DMEM supplemented with 10% FBS, 100 units/ml penicillin, and 100 μg/ml streptomycin. To perform SILAC labeling, cells were cultured in DMEM growth medium supplemented with light (L) isotope-labeled ^12^C_6_-lysine/ ^12^C_6_^14^N_4-_arginine and heavy (H) isotope-labeled ^13^C_6_-lysine/ ^13^C_6_^15^N_4-_arginine (Thermo Fisher Scientific), respectively, for 6 passages. L- and H-labeled cells were maintained in the same growth medium according to the instruction provided by manufacturer.

### Proximity labeling of putative Siglec-E ligands

To perform proximity labeling [16], 10 µg of human immunoglobulin G-Fc fragment (AG714, Merck Millipore) or recombinant Siglec-E with a C-terminal human IgG1-Fc tag (Siglec-E-Fc fusion protein) (551506, Biolegend, San Diego, CA, USA) was pre-incubated with 10 µg of horse peroxidase (HRP)-conjugated anti-Fc antibody at 4 °C for 30 min to form complex. Equal amounts of L- and H-isotope labeled Raw264.7 cells (2 × 10^7^) were then harvested, washed twice with PBS, and resuspended in 1 ml of HBSS containing HRP-Fc complex and HRP-Siglec-E complex, respectively. After incubation at 4 °C for 1 h with rotation, cells were washed twice with ice-cold HBSS and incubated with 10 µM biotin tyramide (SML2135, Sigma) and 10 mM H_2_O_2_ in 1 ml of 20 mM Tris–HCl pH 8.0 buffer containing 140 mM NaCl (TBS) at room temperature for 10 min. Cells were then washed 3 times with ice-cold TBS buffer, followed by lysed in 0.5 ml of RIPA buffer containing protease inhibitor cocktails. After centrifugation at 14,000 xg at 4 °C for 15 min, four hundred µl of supernatant (cell lysate) from each L and H-labeled cells were mixed and incubated with 400 µl of streptavidin-conjugated magnetic beads (88816, Thermo Fisher Scientific) at 4 °C for 2 h with rotation. After washing extensively with RIPA buffer, bound proteins were eluted by 2 × sodium dodecyl sulfate (SDS)-sample buffer. Biotinylated proteins were examined by SDS–polyacrylamide gel electrophoresis (SDS-PAGE) and blotting with HRP-conjugated streptavidin (N100; Thermo Fisher Scientific).

### Proteomic analysis of biotinylated proteins

The eluted biotinylated proteins were subjected to SDS-PAGE with a short (0.5 cm) separating gel. Gel fragment containing proteins were excised, followed by in-gel digestion with trypsin. The tryptic peptides were analyzed on a NanoLC/MS/MS system, which is composed of a nanoUPLC (NanoACQUITY, Waters, Milford, MA,USA) and a high-resolution mass spectrometer (Orbitrap Elite, Thermo Fisher Scientific). The acquired MS raw data were further processed by Proteome Discoverer (v2.2.0.388, Thermo Fisher Scientific) to retrieve the information of protein identification and relative abundance quantification.

### Transient transfection

HEK293T cells originally obtained from ATCC were cultured in DMEM supplemented with 10% FBS, 100 units/ml penicillin, and 100 μg/ml streptomycin. Cells were transfected with HA-SE or Flag-CD36 vector alone, WT HA-SE or HA-SE mutant construct (Siglec-E^R126D^) together with Flag. CD36 construct for 24 h using GenJet™ plus transfection reagent (SL100499, SignaGen. Laboratories, Rockville, MD, USA) according to manufacturer’s instruction. To test the effect of sialyltransferase inhibitor, cells were treated with 200 µM of P-3FAX-Neu5Ac (117405-58-0, Tocris, Ellisville, MO, USA) at 6 h post transfection, and harvested after 66 h incubation in culture. For sialidase treatment, cells transfected for 48 h were rinsed with HBSS, followed by incubation with *Arthrobacter ureafaciens* sialidase as described above for macrophages prior to further experiment. In separate experiment, HEK293T cells were transfected with Flag-Siglec-9 construct together with or without HA-CD36 construct for 48 h, followed by treatment with sialidase for 30 min prior to harvest as described above.

### Immunofluorescence confocal microscopy

HEK293T cells (1 × 10^5^) transfected with FLAG-CD36 together with HA-control, HA-SE, or HA-SE mutant vector as indicated for 24 h were reseeded on cell culture slide and incubated for additional 24 h. Cells were then fixed with 4% paraformaldehyde and permeabilized by 0.2% saponin in PBS at room temperature for 10 min. Following incubation with 3% BSA and 5% goat serum (005–000-121; Jackson ImmunoResearch, West Grove, PA, USA) in PBS at room temperature for 30 min, cells were incubated with rabbit anti-HA-tag (C29F4) (#3724S, Cell Signaling, Danvers, MA, USA) and mouse anti-FLAG M2 (F1804; Sigma) antibodies together at room temperature for 1 h. A separate fixed cell slide incubated with control mouse and rabbit IgGs was served as a negative control. After 3 washes with PBS, cells were incubated with fluorescein isothiocyanate –conjugated goat-anti mouse IgG (GTX26785; GeneTex) and Alexa Fluor 568-conjugated goat-anti-rabbit IgG (A-11011; Invitrogen) to detect FLAG- and HA-antigen–antibody complexes, respectively. After nuclear stain by 4′,6-diamidino-2 phenylindole, cells were visualized by confocal microscope (LSM 510 META, ZEISS) using 63 × oil objective.

### Immunoprecipitation and Western blot analysis

Cells were lysed in buffer containing 50 mM Tris–HCl pH 7.4, 150 mM NaCl, 50 mM NaF, 1 mM Na_3_VO_4,_ 5 mM EDTA, 0.1% SDS, 1% Triton X-100, and protease inhibitor cocktails. Cell lysates were cleared by centrifugation at 14,000 xg for 10 min at 4 °C. To perform immunoprecipitation of endogenous proteins, macrophage cell lysates (~ 1 mg) were incubated with 10 µg of control IgG, anti-Siglec-E antibody (682802, Biolegend) or anti-CD36 antibody (MF3, abcam) as indicated at 4 °C for 18 h with rotation. Protein A/G-conjugated magnetic beads (#88803, Thermo Fisher Scientific) was then added, and incubation continued for additional 3 h at 4 °C. Beads were collected and washed 3 times with lysis buffer, followed by elution of proteins bound to beads with 2 × SDS sample buffer. To perform immunoprecipitation of Flag-CD36 overexpressed in HEK293T cells, cell lysates were prepared and ~ 400 μg of proteins was incubated with 20 μl of anti-FLAG®M2 affinity resin (A2220, Sigma) for 2 h at 4 °C with rotation. Resin was then washed 4 times with the same lysis buffer and bound proteins eluted using 2 × SDS sample buffer. To immunoprecipitate HA-SE or HA-CD36 overexpressed in HEK293T cells, anti-HA affinity resin (A2095, Sigma) was used as described above. To perform Western blot analysis of cell lysates and immunoprecipitated proteins, the following antibodies were used: anti-Siglec-E (AF5806, R&D, Minneapolis, MN, USA), anti-CD36 (AF2519, R&D), anti-phosphoVAV_Y160_ (MAB37861, R&D), anti-VAV antibody (sc8039, Santa Cruz), anti-SHP-1 (#3759, Cell Signaling), anti-HA (#2367S, Cell Signaling), anti-phospho-tyrosine antibody (#05–321, Merck Millipore), anti-GAPDH (GTX100118, Genetex), and anti-Flag M2 (F1804, Sigma) antibodies.

### Lectin and streptavidin blotting

Cell lysates or immunoprecipitated CD36 were subjected to SDS-PAGE and transferred to poly(vinylidene fluoride membranes. After blocking with 2% BSA in PBS containing 0.05% Tween-20 (PBS-T), membranes were incubated with biotin-labeled *Sambucus nigra* lectin (SNA) (B-1305, Vector Laboratories, Burlingame, CA, USA) and *Maackia Amurensis* Lectin II (MALII) (B-1265, Vector Laboratories), which bind to 2,6- and 2,3-linked sialic acids, respectively, in PBS containing 0.1 mM CaCl_2_ at room temperature for 1 h. After 3 washes with PBS, the glycan-lectin complex was detected by HRP-conjugated streptavidin and enhanced chemiluminescence system (RPN2106; GE Healthcare, Uppsala, Sweden).

### Statistical analysis

Quantitative data were presented as mean ± SE of at least 3 independent experiments. Statistical analysis was performed using GraphPad Prism software (v5.03). Statistical difference between two groups was assessed by Student’s t test. One-way ANOVA was used for data more than two groups. *P* < 0.05 was considered statistically significant.

## Results

### Siglec-E deficiency promotes foam cells and necrotic core in atherosclerotic lesion without affecting serum lipid profile in apoE^−/−^ mice

**Fig. 1:**
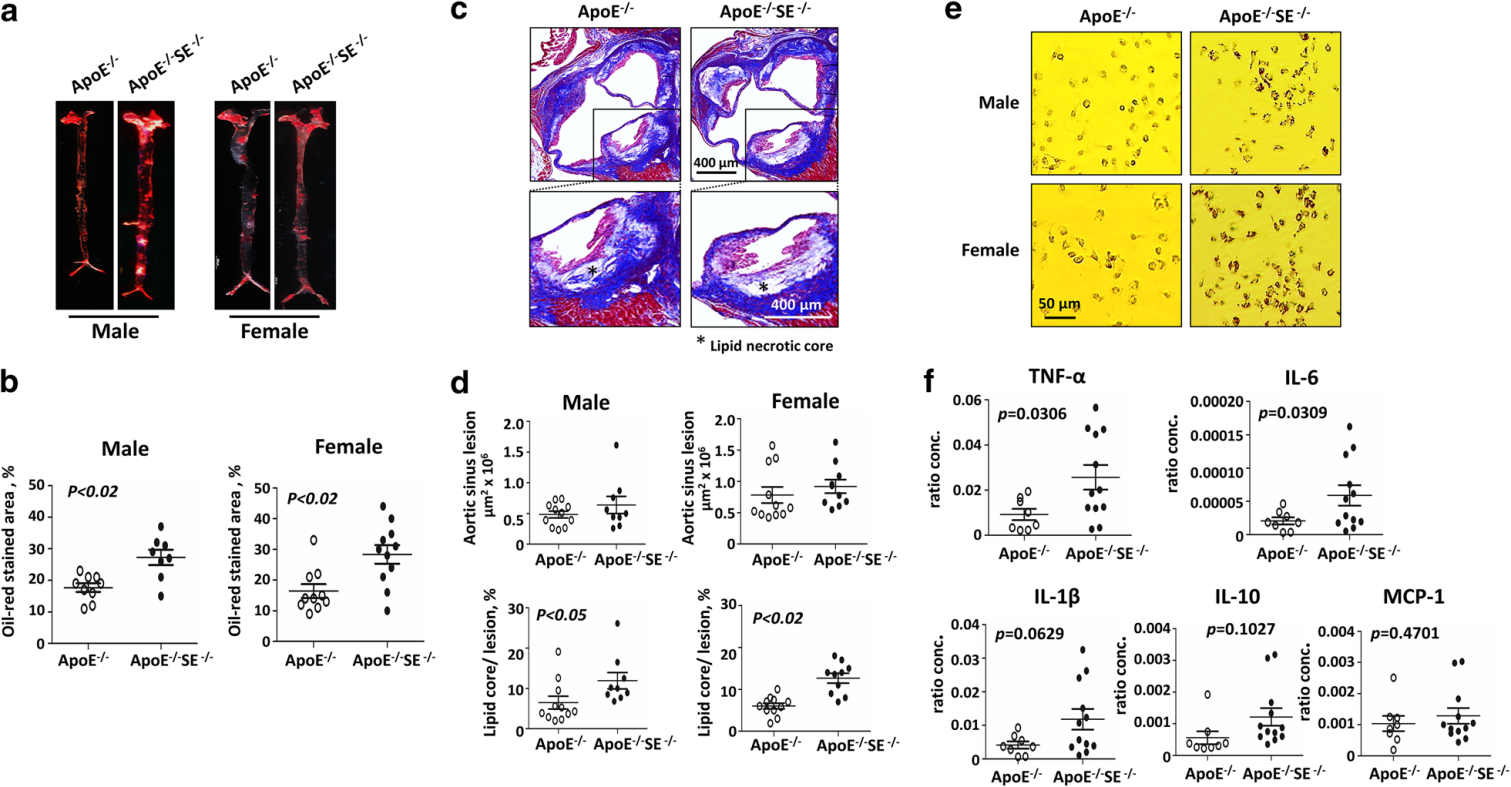
Siglec-E deficiency promotes foam cells and necrotic core in atherosclerotic lesion in apoE^−/−^ mice. ApoE^−/−^ and apoE^−/−^/SE^−/−^ mice were placed on HFD for 3 months. Heart and aortic tissues were harvested (**a**–**d**). **a** The representative images of oil-red dye (ORD) stained aortic tissues. **b** The percentages of ORD stained areas in both groups of mice were quantified. **c** The representative images of Trichrome stained aortic root sections of male mice. **d** The lesion sizes and the percentages of lipid cores in the aortic roots were quantified. **e** Peritoneal macrophages isolated from HFD-fed apoE^−/−^ and apoE^−/−^/SE^−/−^ mice were examined by ORD staining. **f** The mRNA expression levels of indicated cytokines in macrophages were examined by quantitative RT-PCR

To assess the impact of Siglec-E expression on atherosclerosis development, we generated apoE^−/−^ mice with Siglec-E gene deletion (apoE^−/−^/SE^−/−^ mice) for in vivo studies. Both apoE^−/−^ and apoE^−/−^/SE^−/−^ mice (8–10 weeks old) were placed on HFD for 3 months to facilitate lesion formation and their serum lipid profiles were examined. As shown in Additional file 1: Figure S1, the increase of body weight and serum levels of triglyceride, total cholesterol, LDL and HDL were comparable between these two groups of mice in both sexes. Animals were then sacrificed and the lesion formation in the aortic tissues examined by en face ORD staining, which detects the lipid deposition in the aortic wall. As illustrated in Fig. 1a, b, the ORD stained area was significantly larger in apoE^−/−^/SE^−/−^ mice compared to apoE^−/−^ mice irrespective of sex. The trichrome-stained advanced lesions developed in the aortic roots of these mice were also examined. As shown in Fig. 1c, d, the lesion sizes were comparable in apoE^−/−^ and apoE^−/−^/SE^−/−^ mice. Whereas the necrotic core was more prominent in the lesions of apoE^−/−^/SE^−/−^ mice. Likewise, more macrophages identified by staining with F4/80 antibody were present in the lesions of apoE^−/−^/SE^−/−^ mice (Additional file 1: Figure S2a, b). To confirm the increased lipid accumulation in aortic lesions in apoE^−/−^/SE^−/−^mice is resulted from enhanced foam cell formation, the peritoneal macrophages were isolated from HFD-fed apoE^−/−^ and apoE^−/−^/SE^−/−^ mice and stained with ORD. As shown in Fig. 1e, more lipids had accumulated in macrophages from apoE^−/−^/SE^−/−^ mice compared to apoE^−/−^ mice. An experiment was also performed to examine the inflammatory cytokine gene expression profile. As shown in Fig. 1f, the mRNA levels of pro-inflammatory cytokine TNF-α and IL-6 were significantly higher in apoE^−/−^/SE^−/−^ macrophages than apoE^−/−^ counterparts. The level of IL-1β mRNA was also moderately higher in apoE^−/−^/SE^−/−^ macrophages. No significant difference was observed for IL-10 and MCP-1 expressions between the two groups of cells.

### Siglec-E deficiency facilitates modified LDL uptake without affecting cholesterol efflux

**Fig. 2:**
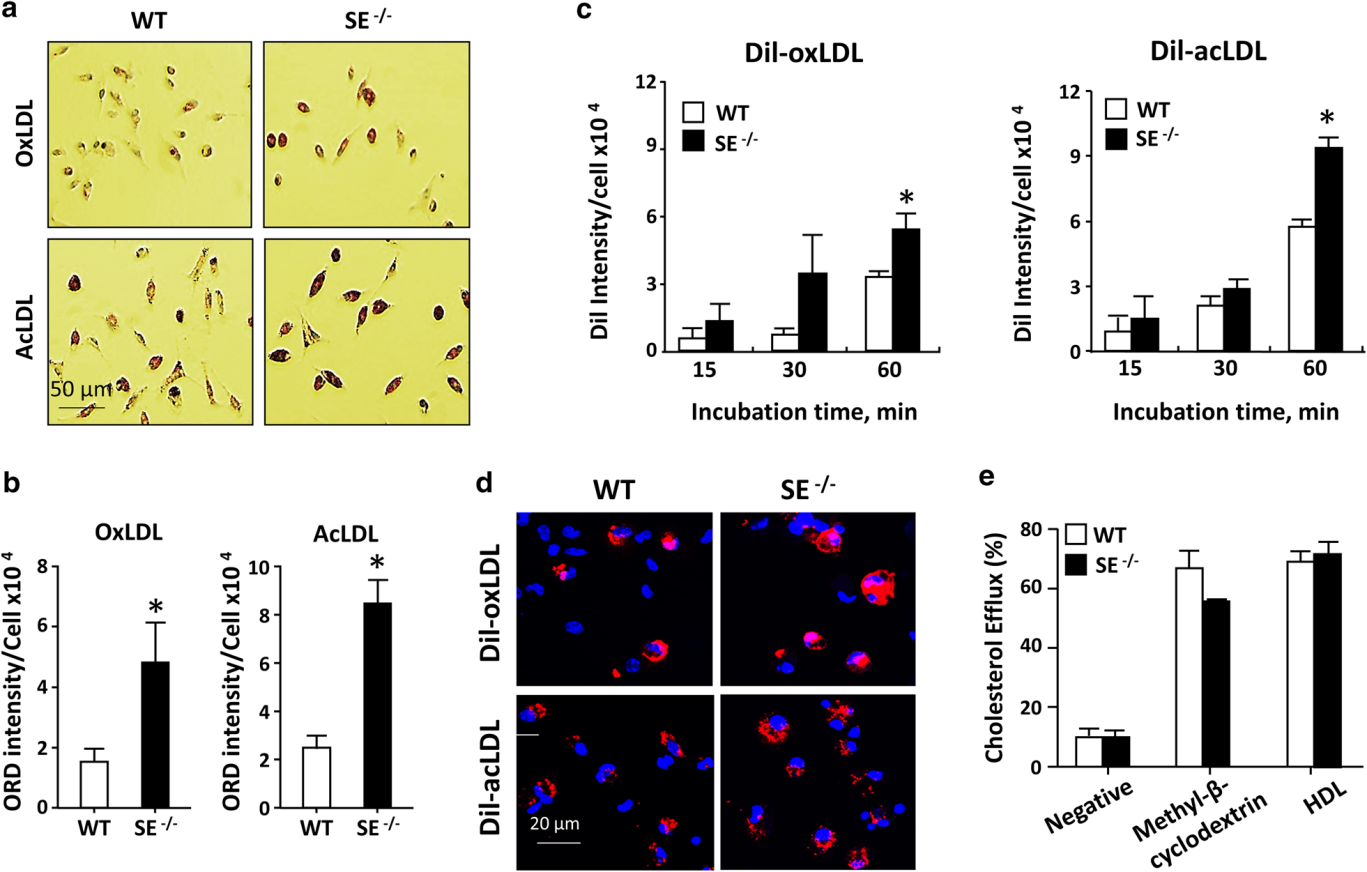
Siglec-E deficiency facilitates modified LDL uptake without affecting cholesterol efflux. Peritoneal macrophages were isolated from WT and SE^−/−^ mice. **a** Macrophages were incubated with 50 µg/ml of oxLDL or acLDL as indicated for 24 h in culture. The accumulation of cholesterols was examined by ORD staining. **b** The quantitative results of ORD intensities. Data shown are the mean ± SE of 3 independent experiments. *P < 0.02 vs WT. **c** Macrophages were incubated with 10 μg/ml of Dil-oxLDL or Dil-acLDL at 37 °C for indicated times. The amounts of uptake in each time point were quantified. Data shown are mean ± SE of 4 independent experiments. *P < 0.03 vs WT. **d** The representative images showing Dil-oxLDL or Dil-acLDL uptake after 1 h incubation. **e** Macrophages preloaded with fluorescently-labeled cholesterol analogue were incubated without (negative) or with indicated agents for 6 h in culture. The percentage of fluorescence intensity in the medium was calculated. Results shown are the mean ± SE of 3 mice in each genotype

To further assess the effect of Siglec-E on foam cell formation, peritoneal macrophages isolated from WT and SE^−/−^ mice were incubated with 50 µg/ml of acLDL or oxLDL in culture. After 24 h, the cholesterol accumulation was examined by ORD staining. As illustrated in Fig. 2a, b, more foam cells were detected in SE^−/−^ macrophages than WT counterparts. Given that the level of intracellular cholesterol is influenced by the balance between modified LDL uptake and cholesterol efflux [17], we then performed experiments to examine the effect of Siglec-E on these two processes. We first examined whether Siglec-E affects the binding of modified LDL. When cells were incubated with 10 ug/ml of fluorescent dye-labeled Dil-acLDL or Dil-oxLDL at 4 °C for 1 h, the quantities of Dil-labeled modified LDL bound on surfaces of both WT and SE^−/−^ macrophages were comparable (Additional file 1: Figure S3a, b), indicating that Siglec-E expression does not affect the binding of modified LDL to their surface receptors on macrophages. However, when the incubation was performed at 37 °C, more Dil-labeled modified LDL accumulated inside SE^−/−^ macrophages after 1 h incubation (Fig. 2c, d). We then performed a cholesterol efflux assay using a fluorescently-labeled cholesterol analogue. As shown in Fig. 2e, the extents of cholesterol efflux induced by methyl-β-cyclodextrin as a positive control or HDL were comparable between WT and SE^−/−^ macrophages. Collectively, the results suggest that the inhibitory effect of Siglec-E on foam cell formation is primarily through downregulating the uptake of modified LDL.

### Identification of putative Siglec-E ligands by proximity labeling method

**Fig. 3:**
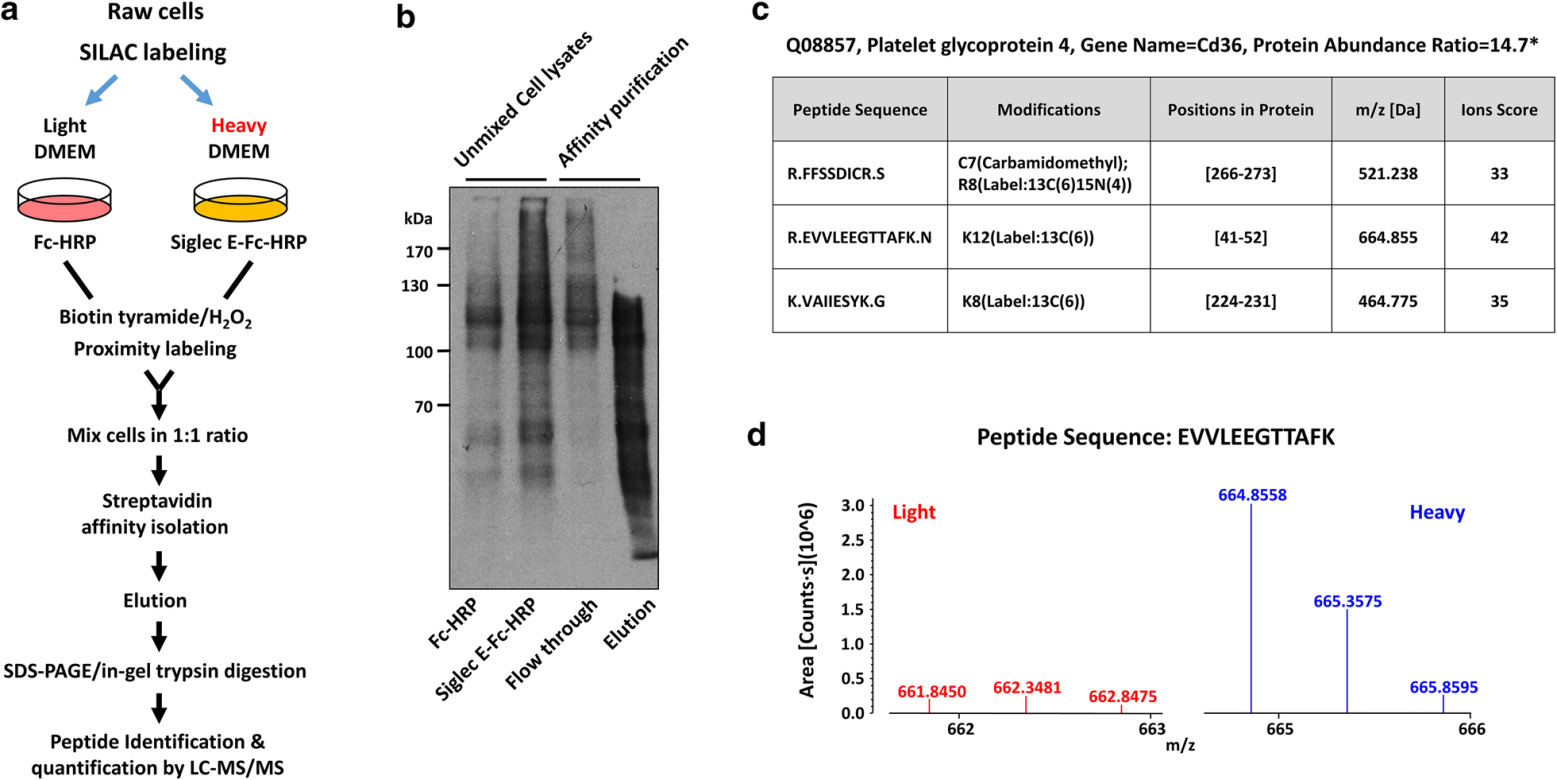
Identification of putative Siglec-E ligands by proximity labeling method. **a** Schematic overview of the procedure to identify Siglec-E ligands. **b** Western blot analysis of biotinylated proteins in cell lysates and affinity-purified fraction. **c** The list of identified CD36 peptides. Protein abundance ratio was calculated from the median of peptide ratios. **d** The Quantification Spectrum of selected peptide (EVVLEEGTTAFK) of CD36

Siglecs can interact with their sialoglycan ligands expressed on the same cells (cis-ligand) or other cells (trans-ligands). To explore the cellular mechanism underlying the effect of Siglec-E on foam cell formation, we adopted the proximity labeling method [16] to identify the putative Siglec-E ligands. As illustrated in Fig. 3a, we first labeled Raw264.7 cells, which are murine macrophage cell line, with light (L) and heavy (H) isotopes for 6 passages using SILAC-labeling technique, and then performed proximity labeling by incubating the L- and H-labeled cells with Fc protein and Siglec-E-Fc fusion protein pre-bound with HRP-conjugated anti-Fc antibody, respectively. Equal amounts of cells were then mixed and cell lysate prepared for the subsequent isolation of biotinylated proteins. Streptavidin blot analysis of the unmixed cell lysates clearly showed that more proteins were biotinylated in cells incubated with Siglec-E-Fc-HRP complex comparing to cells incubated with Fc-HRP control (Fig. 3b). The biotinylated proteins were then isolated and subjected to proteomic analysis. Several proteins with L/H ratio greater than 3.0 were identified as listed in Additional file 1: Table S1. It was found that CD36, which is a class B scavenger receptor responsible for modified LDL uptake in macrophages [18], is one of the putative interacting proteins of Siglec-E. Three unique peptides of CD36 were identified for the quantitative analysis (Fig. 3c). The mass spectrum of an identified peptide was depicted in Fig. 3d.

### Siglec-E interacts with CD36 independent of sialic acid binding activity

**Fig. 4:**
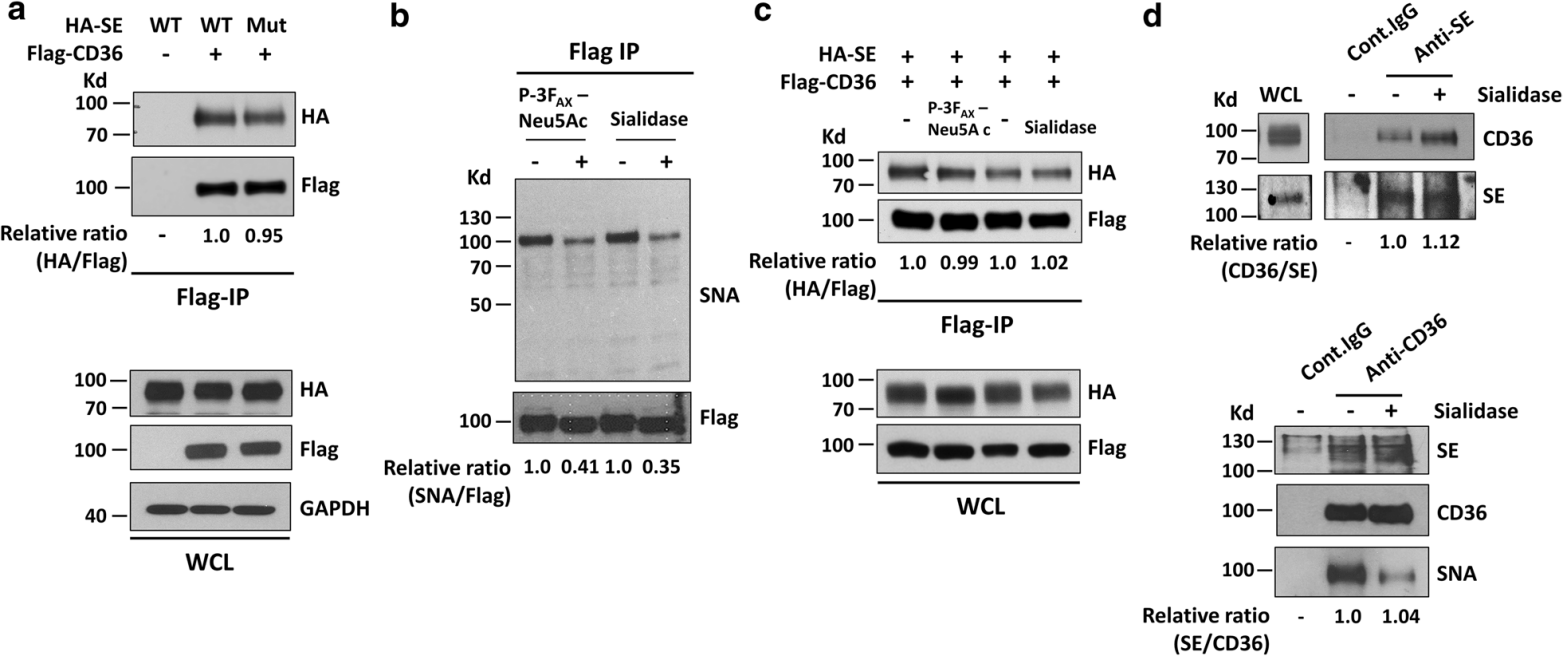
Siglec-E interacts with CD36 independent of sialic acid binding activity. **a** HEK293 T cells were transfected with indicated vectors for 48 h. Cell lysates were prepared and subjected to immunoprecipitation with anti-Flag affinity resin. The expressions of Flag-CD36 and WT and mutant HA-SE protein in whole cell lysates (WCL) and immunoprecipitates were examined by Western blot analysis with indicated antibodies. **b**, **c** HEK293T cells overexpressing Flag-CD36 and WT HA-SE were pretreated with P-3FAX-Neu5Ac for 66 h or with 0.1 unit/ml of sialidase for 30 min at 37 °C prior to immunoprecipitation with anti-Flag affinity resin. Immunoprecipitates were then subjected to SNA lectin blotting (**b**) and Western blot analysis with indicated antibodies (**c**). **d** Peritoneal macrophages isolated from WT mice were treated without or with 0.1 unit/ml of sialidase for 30 min at 37 °C. Cleared cell lysates were subjected to immunoprecipitation with control IgG, anti-Siglec-E or anti-CD36 antibody as indicated. The immunoprecipitates were examined by Western blot analysis with indicated antibodies. The blots of immunoprecipitates were subjected to densitometry analysis and the relative quantitative results were shown under the blots. The value of control group for comparison in each blot was referred to 1

To confirm whether Siglec-E interacts with CD36, we performed transient transfection experiment to express Flag-CD36 together with HA-SE or a HA-SE mutant lacking sialic acid binding activity (Siglec-E^R126D^) [8] in HEK293T cells. As revealed by confocal immunofluorescence microscopy, both Flag-CD36 and HA-SE proteins were coexpressed on the surface of transfected HEK293T cells (Additional file 1: Figure S4). Experiment was then performed to immunoprecipitate CD36 protein using anti-Flag-affinity resin. When the immunoprecipitated proteins were subjected to Western blot analysis, the results showed that both WT and mutant HA-SE were coeluted with Flag-CD36, suggesting that sialic acid is not involved in the interaction between CD36 and Siglec-E (Fig. 4a). To further confirm this observation, we first examined the sialylation status of CD36 by performing lectin blotting. As demonstrated in Additional file 1: Figure S5, the binding signal of SNA to CD36 was much stronger than that of MALII, indicating that CD36 was predominantly modified by α 2, 6-linked sialylation. Treatment with 200 µM of P-3FAX-Neu5Ac, which is a cell permeable sialyltransferase inhibitor [19] for 66 h prior to immunoprecipitation significantly reduced the extent of CD36 sialylation without affecting its interaction with HA-SE (Fig. 4b, c). Likewise, when HEK293T cells overexpressing both proteins were pretreated with *Arthrobacter ureafaciens* sialidase (0.1 unit/ml) at 37 °C for 30 min prior to immunoprecipitation experiment, again it did not affect the interaction between Flag-CD36 and HA-SE (Fig. 4b, c). We also performed experiment with mouse peritoneal macrophages to examine the interaction between endogenous CD36 and Siglec-E. Macrophages were pretreated with or without sialidase for 30 min prior to lysis. Cell lysates were then subjected to immunoprecipitation with control IgG or anti-Siglec-E antibody. As shown in Fig. 4d, CD36 was detected in the immunoprecipitates of Siglec-E from cells with or without sialidase treatment. When the immunoprecipitation was performed with anti-CD36 antibody, Siglec-E was detected in CD36 immunoprecipitate. Again, it was not affected by sialidase treatment as shown in the same figure. Together these results demonstrate that Siglec-E interacts with CD36 independently of sialic acid. To examine whether similar phenomenon is observed with Siglec-9 and CD36, we performed transfection experiment with Flag-Siglec-9 and HA-CD36 constructs in HEK293T cells for 48 h, followed by treatment with sialidase for 30 min as described above prior to immunoprecipitation with anti-HA tag affinity resin. Western blot analysis of the immunoprecipitates again revealed the interaction between Flag-Siglec-9 and HA-CD36 independently of sialic acid (Additional file 1: Figure S6).

### Sialic acid is involved in Siglec-E-mediated inhibition of oxLDL uptake by CD36

**Fig. 5:**
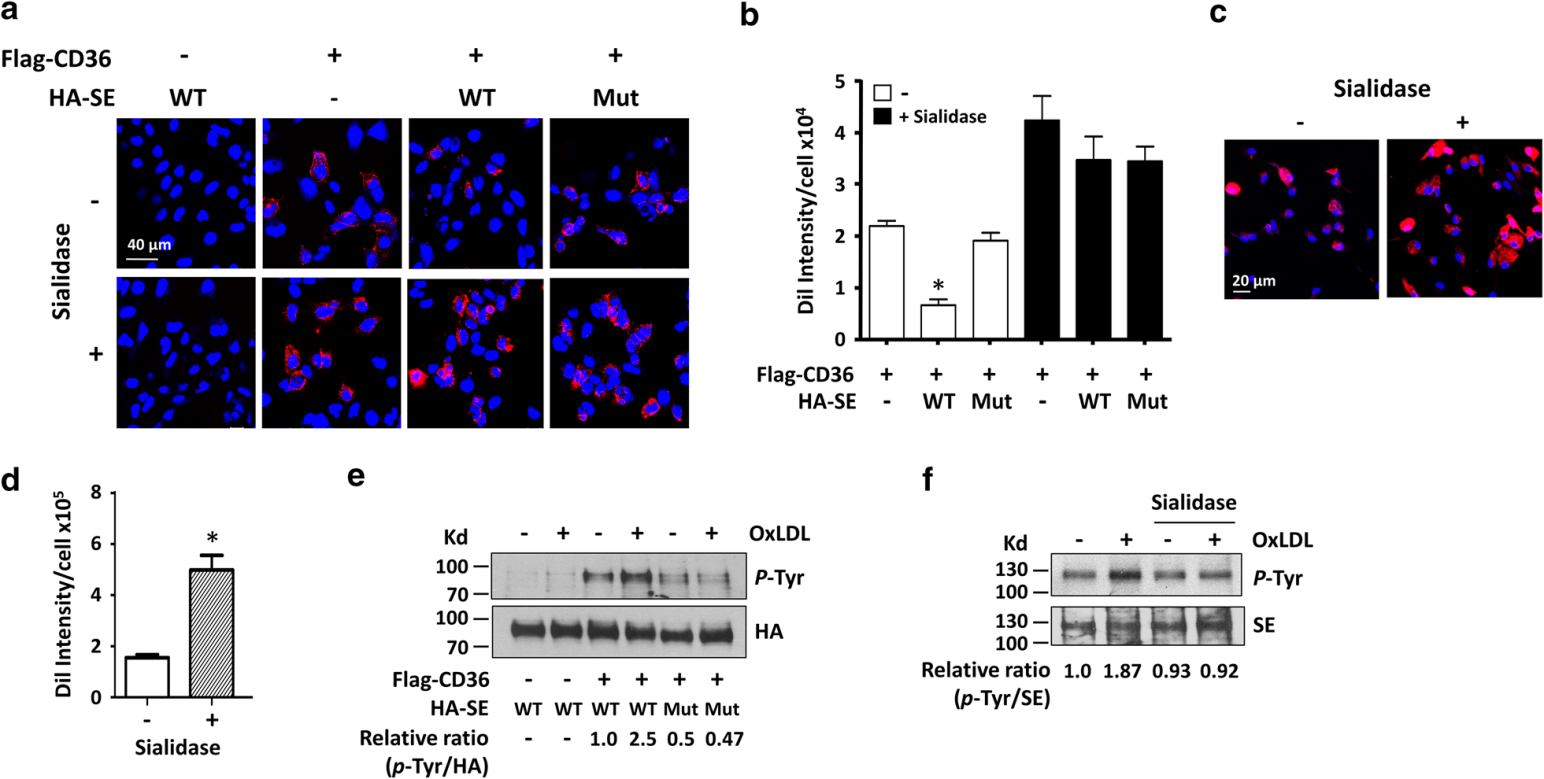
Sialic acid is involved in Siglec-E-mediated inhibition of oxLDL uptake by CD36. **a** HEK293T cells transfected with indicated vectors for 48 h were treated without or with sialidase (0.1 unit/ml) for 30 min at 37 °C, followed by incubation with Dil-oxLDL (5 μg/ml) at 37 °C for 1 h. The intracellular accumulation of Dil-oxLDL was examined by confocal microscopy. **b** The quantitative results of Dil-oxLDL uptake in various groups of cells. Data shown are the mean ± SE of 3 independent experiments. *P < 0.05 vs cells overexpressing Flag-CD36 alone. **c** Peritoneal macrophages were pretreated without or with sialidase, followed by incubation with Dil-oxLDL as described above. Dil-oxLDL uptake was examined by confocal microscopy. **d** The quantitative results of (**c**). Data shown are the mean ± SE of 3 independent experiments. *P < 0.01 vs untreated cells. **e** HEK293T cells transfected with indicated vectors were treated without or with oxLDL (50 μg/ml) for 30 min at 37 °C. Cell lysates were subjected to immunoprecipitation with anti-HA tag affinity resin. The immunoprecipitates were then analyzed by Western blotting with anti-phosphotyrosine and anti-HA antibodies. The relative quantitative results were shown under the blot. **f** WT peritoneal macrophages were pretreated with or without sialidase, followed by incubation without or with oxLDL as described above. Cell lysates were prepared and immunoprecipitation was performed with anti-Siglec-E antibody. The immunoprecipitates were then analyzed by Western blotting with anti-phosphotyrosine and anti-Siglec-E antibodies. The relative quantitative results were shown under the blot

We then examined whether Siglec-E has an impact on CD36-mediated oxLDL uptake. To this end, HEK293T cells overexpressing Flag-CD36 alone or together with WT or mutant HA-SE were incubated with Dil-oxLDL (5 µg/ml) for 1 h. As shown in Fig. 5a, b, intracellular Dil-oxLDL accumulation was evident in cells overexpressing Flag-CD36 but not in cells overexpressing WT HA-SE. Notably, coexpression of WT but not mutant HA-SE significantly reduced Dil-oxLDL accumulation in Flag-CD36 overexpressing cells. The decrease in Flag-CD-36-mediated Dil-oxLDL uptake was not due to the lower surface expression of Flag-CD36 in cells coexpressing WT HA-SE (Additional file 1: Figure S4). When cells were pretreated with sialidase prior to incubation with Dil-oxLDL, Flag-CD36-mediated Dil-oxLDL uptake was markedly increased and not affected by the coexpression of WT or mutant HA-SE (Fig. 5a, b). The impact of desialylation on oxLDL uptake was also demonstrated in macrophages (Fig. 5c, d). Given that binding of Siglec-E with its sialoglycan ligands results in tyrosine phosphorylation of its ITIMS, which is crucial for subsequent recruitment of SHP-1/2 and transducing inhibitory signal [8, 9], we perform further experiment to explore the potential effect of modified LDL on Siglec-E activation. HEK293T cells overexpressing WT HA-SE alone, WT or mutant HA-SE together with Flag-CD36 as indicated were treated with oxLDL (50 µg/ml) for 30 min, and the level of phospho-tyrosine of HA-SE was assessed. As shown in Fig. 5e, oxLDL has no effect on the phosphorylation status of WT HA-SE expressed alone in HEK293T cells. When WT HA-SE-overexpressing cells were incubated with Dil-oxLDL, there was no specific Dil-oxLDL binding on cell surface (Additional file 1: Figure S7), indicating that oxLDL is not a ligand for HA-SE. However, oxLDL treatment enhanced tyrosine phosphorylation of WT but not mutant HA-SE in HEK293T cells coexpressing Flag-CD36 (Fig. 5e). Moreover, sialidase pretreatment reduced oxLDL-induced tyrosine phosphorylation of WT HA-SE (Additional file 1: Figure S8). When we performed the experiment with peritoneal macrophages, again oxLDL induced tyrosine phosphorylation of endogenous Siglec-E, which was abolished by sialidase pretreatment (Fig. 5f). These data demonstrate that the binding of Siglec-E with its sialoglycan ligand following CD36-oxLDL interaction is essential for its negative regulation of CD36-mediated oxLDL uptake.

### Siglec-E downregulates CD36-mediated signaling

**Fig. 6:**
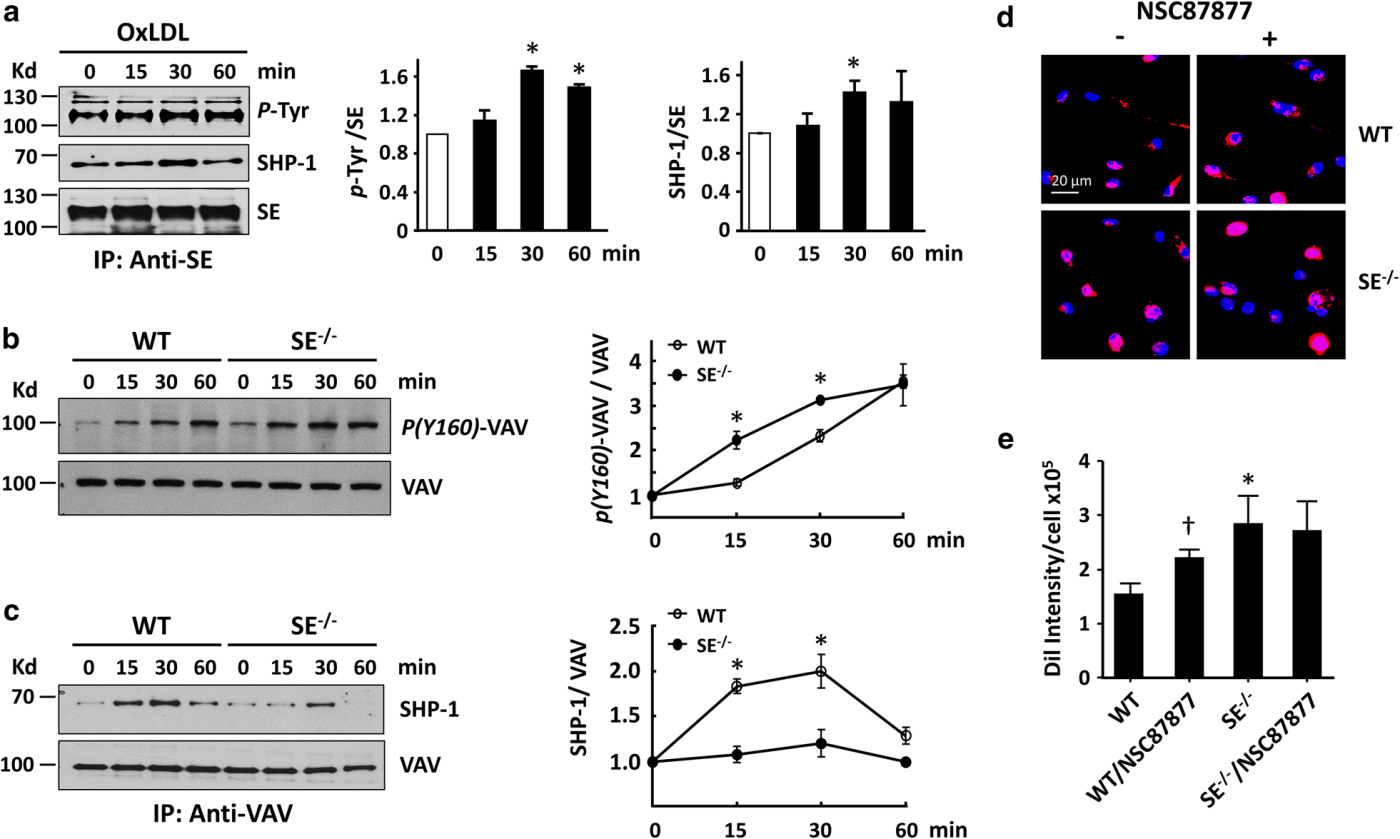
Siglec-E downregulates CD36-mediated signaling. **a** WT peritoneal macrophages were treated with oxLDL (50 μg/ml) for indicated times. Cell lysates were prepared, followed by immunoprecipitation with anti-Siglec E antibody. The immunoprecipitates were then subjected to Western blot analysis with indicated antibodies and analyzed by densitometry. The value at zero time point was referred to 1. The quantitative data are mean ± SE of 3 independent experiments. *P < 0.05 vs zero time point. **b** WT and SE^−/−^ macrophages were treated with oxLDL (50 μg/ml) for indicated times, and the cell lysates were analyzed by Western blotting with anti-phosphoVAV and VAV antibodies and analyzed by densitometry. The value at zero time point was referred to 1. The quantitative data are mean ± SE of 3 independent experiments. *P < 0.05 vs WT group at the same time point. **c** The cell lysates prepared from WT and SE^−/−^ macrophages treated with oxLDL for indicated times as described above were subjected to immunoprecipitation with anti-VAV antibody. The immunoprecipitates were subjected to Western blot analysis with indicated antibodies and analyzed by densitometry. The value at zero time point was referred to 1. The quantitative data showing relative SHP-1/VAV are mean ± SE of 3 independent experiments. *P < 0.05 vs WT group at the same time point. **d** WT and SE^−/−^ macrophages were pretreated with 10 µM of NSC87877 for 30 min in culture, followed by incubation with Dil-oxLDL (10 μg/ml) for another 1 h. The accumulation of intracellular Dil-oxLDL was examined by confocal microscopy. **e** The quantitative results of (**d**). Data shown are the mean ± SE of 3 independent experiments. *P < 0.05 vs untreated WT cells; ^Ɨ^*P* < 0.05 vs untreated WT cells

We then assessed whether oxLDL-induced Siglec-E activation promotes the recruitment and activation of SHP-1 phosphatase to affect CD36 signaling. As shown in Fig. 6a, time course experiment demonstrated that oxLDL induced a transient Siglec-E tyrosine phosphorylation and interaction with SHP-1. Concurrently, oxLDL induced the phosphorylation of VAV, a guanine nucleotide exchange factor implicated in CD36-mediated oxLDL uptake and foam cell formation [20, 21], in both WT and SE^−/−^ macrophages (Fig. 6b). Coimmunoprecipitation experiment again revealed the increase of interaction between VAV with SHP-1 following oxLDL treatment. Notably, the interaction between VAV and SHP-1 was more prominent in WT macrophages (Fig. 6c), which was correlated with lower phosphorylation of VAV in WT cells comparing to SE^−/−^ counterparts (Fig. 6b), supporting the negative regulation of CD36 downstream signaling by Siglec-E/SHP-1 axis. To further evaluate the effect of Siglec-E/SHP-1-mediated inhibitory signaling on oxLDL uptake, macrophages were pretreated with SHP-1 inhibitor NSC87877 [15] prior to incubation with Dil-oxLDL for 1 h. As shown in Fig. 6d, e, SHP-1 inhibition significantly increased Dil-oxLDL uptake in WT macrophages but not on SE-/- macrophages. These findings support the notion that Siglec-E suppresses oxLDL uptake and foam cell formation by attenuating CD36-induced downstream signaling in macrophages.

## Discussion

Sialic acids have been implicated in several aspects of pathophysiology underlying atherosclerosis. Earlier studies have revealed the positive correlation between high plasma total sialic acids and the risk of coronary heart disease [22–24]. Moreover, the sialic acid content is lower in LDL isolated from patients with coronary heart disease [25]. The content of sialic acid is reduced in oxidative modified LDL [26], and LDL de-sialylation results in more accumulation of lipid in cells [27]. These findings suggest the potential involvement of sialic acid in the regulation of modified LDL uptake and cholesterol accumulation in macrophages. Besides, sialic acids expressed on the surface of leukocytes play vital roles in cell recognition and adhesion, cell–cell interaction and signal transduction [29]. Siglecs are widely expressed on cells of immune system and modulate immune functions via sialic acid-Siglec axis [5]. It has been shown that the expression of Siglec-1, which lacks the ITIM motifs observed in many Siglecs, is increased on circulating monocytes and correlated with the severity of coronary artery disease in human patients [29]. Down regulation of Siglec-1 reduces oxLDL uptake in macrophages in vitro and attenuates atherosclerosis in apoE^−/−^ mice [30]. A recent study also showed that Siglec-G expression on B-1 cells promotes atherosclerosis through inhibiting the production of oxLDL-specific IgM, which blocks the uptake of oxLDL by macrophages [31]. Whether other Siglecs have roles in the pathogenesis of atherosclerosis warrants further investigation.

**Fig. 7:**
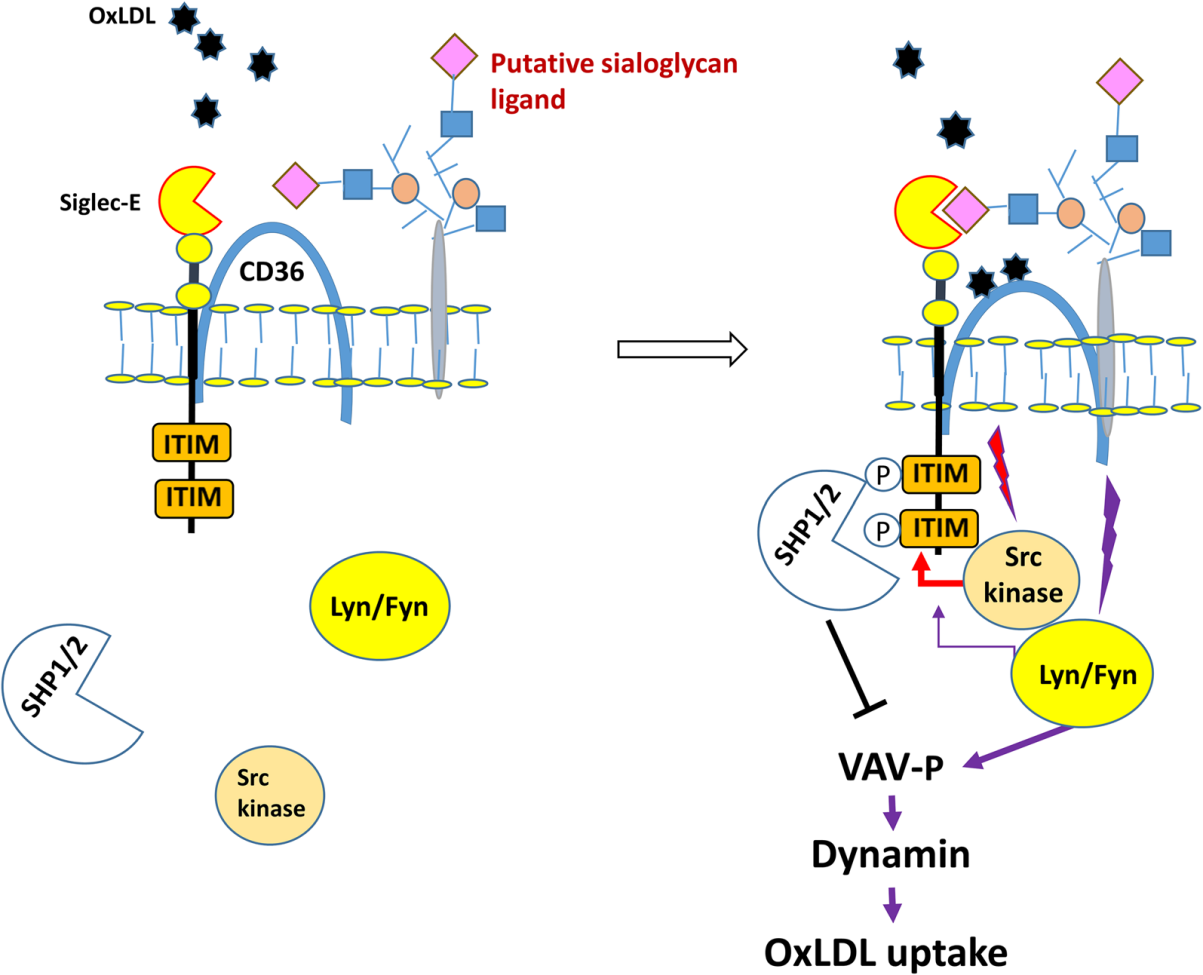
Schematic diagram illustrating Siglec-E-induced negative signaling on CD36-mediated oxLDL uptake on macrophages. Siglec-E physically interacts with CD36 through their polypeptides in resting state. When oxLDL binds to CD36, oxLDL-CD36 complex elicits the recruitment of a putative surface protein containing sialoglycan to interact with Siglec-E and activate Siglec-E/SHP-1 axis, which in turn inhibits CD36 downstream signaling for oxLDL uptake

In the present study, we showed that Siglec-E deficiency accelerates lesion formation and plaque vulnerability in apoE^−/−^ mice. Foam cell formation and the inflammatory response were significantly enhanced in macrophages isolated from apoE^−/−^/SE^−/−^ mice compared to apoE^−/−^ mice. We further demonstrated that Siglec-E expression suppressed modified LDL uptake but not cholesterol efflux in macrophages. Siglec-mediated inhibition on cellular uptake was also observed for CD33 (Siglec-3) which inhibits uptake and clearance of amyloid beta 42 by microglia and is implicated in exacerbation of Alzheimer’s disease [32, 33]. To explore the molecular details how Siglec-E impacts modified LDL uptake, we conducted SILAC in conjunction with proximity labeling to identify CD36 as a potential Siglec-E ligand. CD36 is a highly glycosylated protein expressed on macrophages to mediate modified LDL uptake [34]. We confirmed the interaction between SE and CD36 in HEK293T cells overexpressing these two proteins and in macrophages as well. A recent study by Kawecki et al. demonstrated that membrane neuraminidase 1 (NEU1) interacts with CD36 to modulate its sialylation level and oxLDL uptake in macrophages [35]. Consistent with their report, we found that CD36 is predominantly modified by α 2, 6-linked sialylation. Interestingly, sialic acid was not involved in the interaction between CD36 and Siglec-E. A similar phenomenon has been reported for CD22/Siglec-2 which interacts with CD45 and surface IgM independent of surface sialylation on B cells [36]. Nevertheless, sialic acid binding is essential for CD22 function as a negative regulator of B cell receptor signaling [37, 38]. To explore whether Siglec-E-mediated inhibition of modified LDL uptake by CD36 requires sialic acid binding, we performed further experiments to compare Dil-oxLDL uptake in HEK293T cells overexpressing WT and mutant Siglec-E together with CD36. Similar to previous findings by others [39, 40], CD36 expression alone promotes Dil-oxLDL uptake in HEK293T cells. We showed that the extent of intracellular Dil-oxLDL accumulation was significantly reduced by coexpression of WT but not mutant Siglec-E lacking sialic acid binding activity. Notably, sialidase treatment significantly enhanced the uptake of Dil-oxLDL in CD36-expressing HEK293T cells regardless of whether WT or mutant Siglec-E was coexpressed. Sialidase treatment not only affected the availability of sialoglycan ligands required for Siglec-E activation, it also caused desialylation of CD36 which has been shown to increase CD36-mediated oxLDL uptake [35]. Under this condition, it is conceivable that the coexpression of Siglec-E would not have significant impact on the enhanced oxLDL uptake-mediated by CD36. We also observed the promoting effect of desialylation on oxLDL uptake in macrophages. These findings highlight the importance of cell surface sialylation status on foam cell formation. OxLDL failed to bind and induce tyrosine phosphorylation of Siglec-E expressed alone in HEK293T cells, indicating that oxLDL does not serve as a ligand for Siglec-E. Whereas it promoted WT but not mutant Siglec-E phosphorylation in cells coexpressing CD36. Likewise, desialylation abolished oxLDL-induced Siglec-E phosphorylation in macrophages. These observations suggest that the binding of Siglec-E with a putative sialoglycan ligand presented on a surface protein, which is likely recruited and complexed with oxLDL-bound CD36, is vital to elicit Siglec-E activation as depicted in Fig. 7. Further studies are needed to address this possibility. Interestingly, we noticed there was an increase in the basal phosphorylation of Siglec-E in cells coexpressing CD36 even without oxLDL treatment. Since CD36 is known to bind many different ligands, including fatty acids and other substances in serum [41], it is likely that CD36 can be activated by serum present in the culture medium, resulting in the recruitment of the putative sialoglycan ligand and subsequent Siglec-E phosphorylation to certain extent in the absence of oxLDL. Notably, CD36 coexpression also induced a modest phosphorylation of mutant Siglec-E, which is much less than that of WT Siglec-E. We speculate that the activation of Lyn/Fyn tyrosine kinase following oxLDL-CD36 interaction would also promote phosphorylation of ITIM motifs of Siglec-E to certain degree irrespective of binding with sialoglycan ligand.

It is documented that phosphorylation of VAV following oxLDL binding with CD36 transactivates the downstream small G proteins such as rac/rho and dynamin to mediate the endocytosis of oxLDL [20, 21]. Previous studies have shown that SHP-1-mediated VAV dephosphorylation takes part in FAS-mediated cell death signaling in B cells [42] and inhibition of NK cell-mediated cellular cytotoxicity [43]. Therefore, to further understand how Siglec-E affects CD36-mediated oxLDL uptake in macrophages, we explored the potential impact of Siglec-E/SHP-1 axis on VAV phosphorylation. Co-immunoprecipitation experiment demonstrated that oxLDL treatment induced a transient recruitment of SHP-1 by Siglec-E in macrophages, indicating the activation of Siglec-E/SHP-1 axis by oxLDL. Moreover, oxLDL induced a transient interaction between SHP-1 and VAV, which was more prominent in WT macrophages comparing to SE^−/−^ counterparts. Concurrently, the level of VAV phosphorylation following oxLDL treatment was substantially lower in WT macrophages. The impact of SHP-1 on the modulation of oxLDL uptake was further demonstrated by the experiment showing that SHP-1 inhibition significantly increased Dil-oxLDL uptake in WT macrophages but not in SE^−/−^ macrophages. Collectively, these findings support the significance of crosstalk between Siglec-E and CD36 signalings in the regulation of CD36-mediated oxLDL uptake and foam cell formation.

## Conclusions

The present study provides both in vitro and in vivo evidence to demonstrate the inhibitory function of Siglec-E in the regulation of foam cell formation in the course of atherosclerosis. Although sialic acid is not required for the physical interaction of Siglec-E with CD36, it is pivotal for eliciting Siglec-E negative signaling following oxLDL binding with CD36. A previous study has shown that NEU1 is highly expressed in the macrophages of human atherosclerotic plaques [44]. It is conceivable that the increased desialylation would result in the exacerbation of the uptake and accumulation of cholesterol and inflammatory response in macrophages. A recent study has shown that nanoparticles decorated with sialic acids are effective in promoting Siglec-E-mediated anti-inflammatory response and abrogate sepsis and acute respiratory distress syndrome in animals [45]. Whether similar approach can be taken to augment the activation of Siglec-E/SHP-1 axis to ameliorate atherosclerosis warrants further investigation.

## Supplementary Information

**Additional file 1.** Additional table and figures.

## Data Availability

All data generated and analyzed during the current study are included in this published article and its supplementary information files.

## Abbreviations

AcLDL: Acetylated LDL
BSA: Bovine serum albumin
Dil: 1,1′-Dioctadecyl-3,3,3′,3′-tetramethylindocarbocyanine perchlorate
DMEM: Dulbecco's modified Eagle medium
EDTA: Ethylenediaminetetraacetic acid
FBS: Fetal bovine serum
HBSS: Hank’s balanced saline solution;
HDL: High-density lipoprotein
HFD: High-fat diet
HRP: Horse peroxidase
IL: Interleukin
ITIMS: Immunoreceptor tyrosine-based inhibitory motifs
LDL: Low-densitylipoprotein
MALII: *Maackia Amurensis* Lectin II
MCP-1: Monocyte chemoattractant protein 1
ORD: Oil-red dye
OxLDL: Oxidized LDL; PAGE, polyacrylamide gel electrophoresis
PBS: Phosphate-buffered saline
PCR: Polymerase chain reaction
SDS: Sodium dodecyl sulfate
SE: Siglec-E
SHP: Src homology region 2 domain-containing phosphatase
SILAC: Stable isotope labeling using amino acids in cell culture;
SNA: *Sambucus nigra* Lectin
TNF-α: Tumor necrosis factor-α
VSMC: Vascular smooth muscle cells
WT: Wild type

## References

[1] Finegold JA, Asaria P, Francis DP (2013). Mortality from ischaemic heart disease by country, region, and age: statistics from World Health Organisation and United Nations. Int J Cardiol.

[2] Libby P (2002). Inflammation in atherosclerosis. Nature.

[3] Hansson GK (2005). Inflammation, atherosclerosis, and coronary artery disease. N Engl J Med.

[4] Bentzon JF, Otsuka F, Virmani R, Falk E (2014). Mechanisms of plaque formation and rupture. Circ Res.

[5] Macauley MS, Crocker PR, Paulson JC (2014). Siglec-mediated regulation of immune cell function in disease. Nat Rev Immunol.

[6] Ando M, Tu W, Nishijima K, Iijima S (2008). Siglec-9 enhances IL-10 production in macrophages via tyrosine-based motifs. Biochem Biophys Res Commun.

[7] Laubli H, Pearce OM, Schwarz F, Siddiqui SS, Deng L, Stanczak MA, Deng L, Verhagen A, Secrest P, Lusk C, Schwartz AG, Varki NM, Bui JD, Varki A (2014). Engagement of myelomonocytic Siglecs by tumor-associated ligands modulates the innate immune response to cancer. Proc Natl Acad Sci USA.

[8] McMillan SJ, Sharma RS, McKenzie EJ, Richards HE, Zhang J, Prescott A, Crocker PR (2013). Siglec-E is a negative regulator of acute pulmonary neutrophil inflammation and suppresses CD11b beta2-integrin-dependent signaling. Blood.

[9] Schwarz F (2015). Siglec receptors impact mammalian lifespan by modulating oxidative stress. Elife.

[10] Nakashima Y, Plump AS, Raines EW, Breslow JL, Ross R (1994). ApoE-deficient mice develop lesions of all phases of atherosclerosis throughout the arterial tree. Arterioscler Thromb.

[11] Zhang X, Goncalves R, Mosser DM (2008). The isoaltion and characterization of murine macrophages. Curr Protoc Immunol.

[12] Proudfoot D, Davies JD, Skepper JN, Weissberg PL, Shanahan CM (2002). Acetylated low lowdensity lipoprotein stimulates human vascular smooth muscle cell calcification by promoting osteoblastic differentiation and inhibiting phagocytosis. Circulation.

[13] Steinbrecher UP (1987). Oxidation of human low density lipoprotein results in derivatization of lysine residues of apolipoprotein B by lipid peroxidie decomposition products. J Biol Chem.

[14] Chen Y, Kennedy DJ, Ramakrishnan DP, Yang M, Huang W, Li Z, Xie Z, Chadwick AC, Sahoo D, Silverstein RL (2015). Oxidized LDL-bound CD36 recruits an Na+/K+-ATPase-Lyn complex in macrophages that promotes atherosclerosis. Sci Signal.

[15] Chen L, Sung SS, Yip ML, Lawrence HR, Ren Y, Guida WC, Sebti SM, Lawrence NJ, Wu J (2006). Discovery of a novel Shp2 protein tyrosine phosphatase inhibitor. Mol Pharmacol.

[16] Chang L, Chen YJ, Fan CY, Tang CJ, Chen YH, Low PY, Ventura A, Lin CC, Chen YJ, Angata T (2017). Identification of siglec ligands using a proximity labeling method. J Proteome Res.

[17] Yu XH, Fu YC, Zhang DW, Yin K, Tang CK (2013). Foam cells in atherosclerosis. Clin Chim Acta.

[18] Park YM (2014). CD36, a scavenger receptor implicated in atherosclerosis. Exp Mol Med.

[19] Rillahan CD, Antonopoulos A, Lefort CT, Sonon R, Azadi P, Ley K, Dell A, Haslam SM, Paulson JC (2012). Global metabolic inhibitors of sialyl- and fucosyltransferases remodel the glycome. Nat Chem Biol.

[20] Rahaman SO, Swat W, Febbraio M, Silverstein RL (2011). Vav family Rho guanine nucleotide exchange factors regulate CD36-mediated macrophage foam cell formation. J Biol Chem.

[21] Rahaman SO, Zhou G, Silverstein RL (2011). Vav protein guanine nucleotide exchange factor regulates CD36 protein-mediated macrophage foam cell formation via calcium and dynamin-dependent processes. J Biol Chem.

[22] Råstam L, Lindberg G, Folsom AR, Burke GL, Nilsson-Ehle P, Lundblad A (1996). Association between serum sialic acid concentration and carotid atherosclerosis measured by B-mode ultrasound. The ARIC Investigators. Atherosclerosis risk in communities study. Int J Epidemiol.

[23] Gokmen SS, Kilicli G, Ozcelik F, Ture M, Gulen S (2002). Association between serum total and lipid-bound sialic acid concentration and the severity of coronary atherosclerosis. J Lab Clin Med.

[24] Gopaul KP, Crook MA (2006). Sialic acid: a novel marker of cardiovascular disease?. Clin Biochem.

[25] Ruelland A, Gallou G, Legras B, Paillard F, Cloarec L (1993). LDL sialic acid content in patients with coronary artery disease. Clin Chim Acta.

[26] Oztürk Z, Sönmez H, Görgün FM, Ekmekçi H, Bilgen D, Ozen N, Sözer V, Altuğ T, Kökoğlu E (2007). The relationship between lipid peroxidation and LDL desialylation in experimental atherosclerosis. Toxicol Mech Methods.

[27] Orekhov AN, Tertov VV, Sobenin IA, Smirnov VN, Via DP, Guevara J, Gotto AM, Morrisett JD (1992). Sialic acid content of human low density lipoproteins affects their interaction with cell receptors and intracellular lipid accumulation. J Lipid Res.

[29] Varki A, Gagneux P (2012). Multifarious roles of sialic acids in immunity. Ann N Y Acad Sci.

[29] Xiong YS, Zhou YH, Rong GH, Wu WL, Liang Y, Yang ZX, Geng HL, Zhong RQ (2009). Siglec-1 on monocytes is a potential risk marker for monitoring disease severity in coronary artery disease. Clin Biochem.

[30] Xiong YS, Wu AL, Mu D, Yu J, Zeng P, Sun Y, Xiong J (2017). Inhibition of siglec-1 by lentivirus mediated small interfering RNA attenuates atherogenesis in apoE-deficient mice. Clin Immunol.

[31] Gruber S, Hendrikx T, Tsiantoulas D, Ozsvar-Kozma M, Göderle L, Mallat Z, Witztum JL, Shiri-Sverdlov R, Nitschke L, Binder CJ (2016). Sialic acid-binding immunoglobulin-like lectin G promotes atherosclerosis and liver inflammation by suppressing the protective functions of B-1 cells. Cell Rep.

[32] Griciuc A, Serrano-Pozo A, Parrado AR, Lesinski AN, Asselin CN, Mullin K, Hooli B, Choi SH, Hyman BT, Tanzi RE (2013). Alzheimer's disease risk gene CD33 inhibits microglial uptake of amyloid beta. Neuron.

[33] Bhattacherjee A, Rodrigues E, Jung J, Luzentales-Simpson M, Enterina JR, Galleguillos D, St Laurent CD, Nakhaei-Nejad M, Fuchsberger FF, Streith L, Wang Q, Kawasaki N, Duan S, Bains A, Paulson JC, Rademacher C, Giuliani F, Sipione S, Macauley MS (2019). Repression of phagocytosis by human CD33 is not conserved with mouse CD33. Commun Biol.

[34] Hoosdally SJ, Andress EJ, Wooding C, Martin CA, Linton KJ (2009). The Human Scavenger Receptor CD36: glycosylation status and its role in trafficking and function. J Biol Chem.

[35] Kawecki C, Bocquet O, Schmelzer CEH, Heinz A, Ihling C, Wahart A, Romier B, Bennasroune A, Blaise S, Terryn C, Linton KJ, Martiny L, Duca L, Maurice P (2019). Identification of CD36 as a new interaction partner of membrane NEU1: potential implication in the pro-atherogenic effects of the elastin receptor complex. Cell Mol Life Sci.

[36] Zhang M, Varki A (2004). Cell surface sialic acids do not affect primary CD22 interactions with CD45 and surface IgM nor the rate of constitutive CD22 endocytosis. Glycobiology.

[37] Jin L, McLean PA, Neel BG, Wortis HH (2002). Sialic acid binding domains of CD22 are required for negative regulation of B cell receptor signaling. J Exp Med.

[38] Kelm S, Gerlach J, Brossmer R, Danzer CP, Nitschke L (2002). The ligand-binding domain of CD22 is needed for inhibition of the B cell receptor signal, as demonstrated by a novel human CD22-specific inhibitor compound. J Exp Med.

[39] Amézaga N, Sanjurjo L, Julve J, Aran G, Pérez-Cabezas B, Bastos-Amador P, Armengol C, Vilella R, Escolà-Gil JC, Blanco-Vaca F, Borràs FE, Valledor AF, Sarrias MR (2014). Human scavenger protein AIM increases foam cell formation and CD36-mediated oxLDL uptake. J Leukoc Biol.

[40] Jay AG, Chen AN, Paz MA, Hung JP, Hamilton JA (2015). CD36 binds oxidized low density lipoprotein (LDL) in a mechanism dependent upon fatty acid binding. J Biol Chem.

[41] Febbraio M, Hajjar DP, Silverstein RL (2001). CD36: a class B scavenger receptor involved in angiogenesis, atherosclerosis, inflammation, and lipid metabolism. J Clin Invest.

[42] Koncz G, Kerekes K, Chakrabandhu K, Hueber AO (2008). Regulating Vav1 phosphorylation by the SHP-1 tyrosine phosphatase is a fine-tuning mechanism for the negative regulation of DISC formation and Fas-mediated cell death signaling. Cell Death Differ.

[43] Stebbins CC, Watzl C, Billadeau DD, Leibson PJ, Burshtyn DN, Long EO (2003). Vav1 dephosphorylation by the tyrosine phosphatase SHP-1 as a mechanism for inhibition of cellular cytotoxicity. Mol Cell Biol.

[44] Sieve I, Ricke-Hoch M, Kasten M, Battmer K, Stapel B, Falk CS, Leisegang MS, Haverich A, Scherr M, Hilfiker-Kleiner D (2018). A positive feedback loop between IL-1beta, LPS and NEU1 may promote atherosclerosis by enhancing a pro-inflammatory state in monocytes and macrophages. Vascul Pharmacol.

[45] Spence S (2015). Targeting Siglecs with a sialic acid-decorated nanoparticle abrogates inflammation. Sci. Transl. Med.

