# Supplementary material for: Siglec-E retards atherosclerosis by inhibiting CD36-mediated foam cell formation
Source: J Biomed Sci. 2021 Jan 5;28:5. doi: 10.1186/s12929-020-00698-z (PMC7784283; doi:10.1186/s12929-020-00698-z)
Supplement: Supplementary file 1 — Additional file 1. Additional table and figures. [file 12929_2020_698_MOESM1_ESM.pdf]

SUPPLEMENTAL MATERIALS

Table S1. The list of Siglec E-Fc-proximity labeled membrane proteins identified from SILAC-base quantitative proteomic analysis with heavy/light ratio greater than 3.0

Protein ID	UniProt	No. of Peptides identified	Ratio (Heavy/Light)
Metal transporter CNNM4	Q69ZF7	2	100
Platelet glycoprotein 4 (CD36)	Q08857	3	14.7
Monocarboxylate transporter 4	P57787	7	4.07
Integrin alpha-M	P05555	30	3.93
Choline transporter-like protein 1	Q6X893	5	3.76
Scavenger receptor class B member 1	Q61009	8	3.60
Sodium bicarbonate cotransporter 3	Q8BTY2	15	3.52
Allergin-1	Q3TB92	4	3.31
Transmembrane protein 206	Q9D771	5	3.21
CD276 antigen	Q8VE98	4	3.16
Large neutral amino acids transporter small subunit 1	Q9Z127	4	3.15
Low-density lipoprotein receptor	P35951	27	3.14
Semaphorin-4D	O09126	24	3.12
Serine incorporator 3	Q9QZI9	2	3.10
Tumor necrosis factor receptor superfamily member 5	P27512	2	3.06

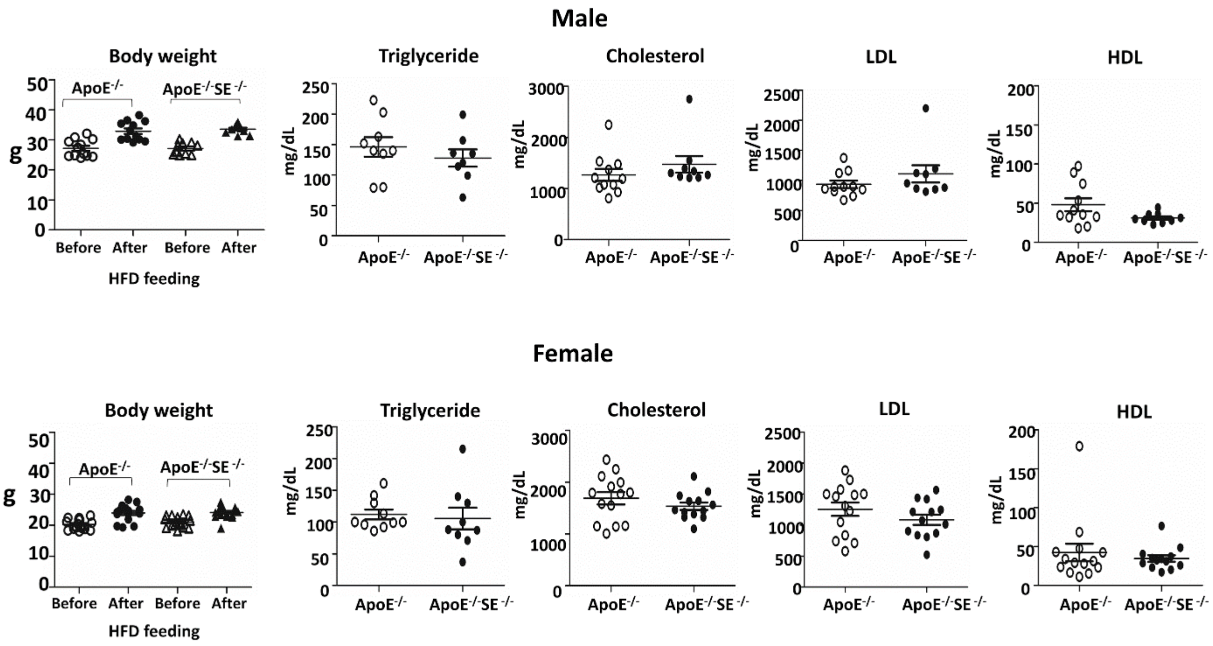

Figure S1. Siglec-E deficiency does not affect body weight gain and lipid profile of apoE$^{-/-}$ mice after 3 months of HFD feeding. ApoE$^{-/-}$ and apoE$^{-/-}$/SE$^{-/-}$ mice (8-10 weeks old) of both sexes were placed on HFD containing 1.25% cholesterol for 12 weeks. Body weights determined before and after HFD feeding. The serum triglyceride, cholesterol, LDL, and HDL levels were determined at the end of HFD feeding.

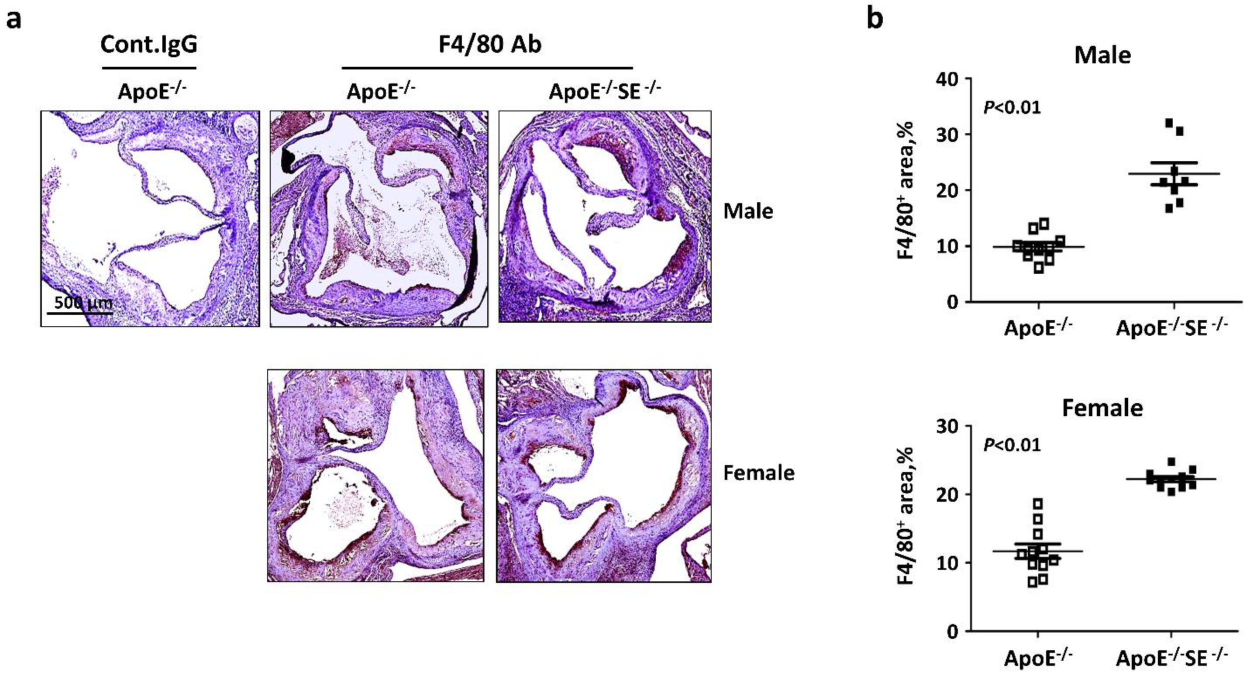

Figure S2. Siglec-E deficiency increases macrophage infiltration in lesions of aortic roots in apoE$^{-/-}$ mice after 3 months of HFD feeding. ApoE$^{-/-}$ and apoE$^{-/-}$/SE$^{-/-}$ mice (8-10 weeks old) of both sexes were placed on HFD containing 1.25% cholesterol for 12 weeks. (a) Representative images of aortic root sections subjected to immunostaining with control IgG or antibody against F4/80 as indicated. (b) The areas with F4/80^{+}-macrophages were quantified and expressed as percentages of lesions.

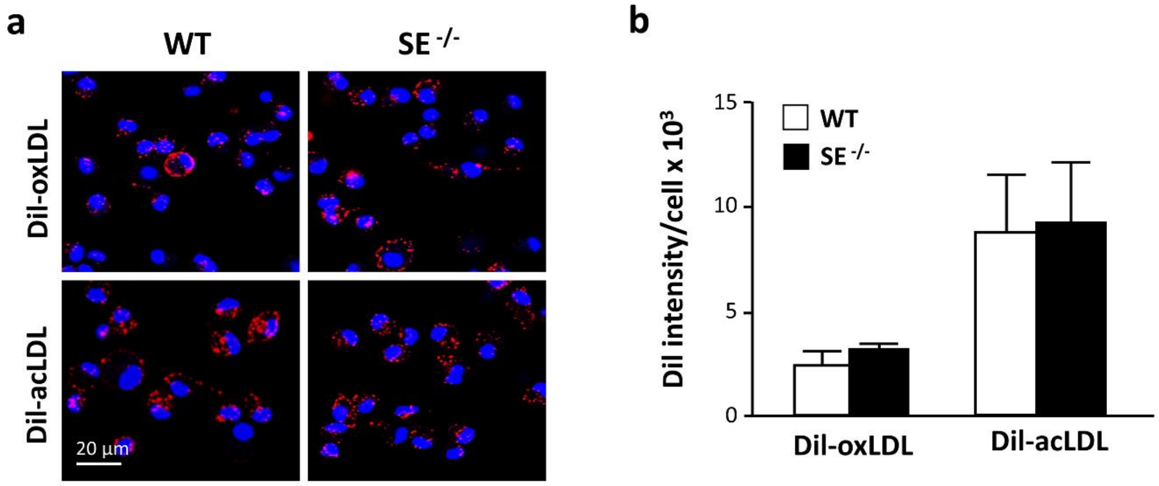

Figure S3. Siglec-E deficiency does not affect binding of modified LDL to macrophages. (a) Peritoneal macrophages isolated from WT and $SE^{-/-}$ mice were incubated with 10 µg/ml of Dil-labeled modified LDL as indicated at 4°C for 1 h. Cells were then washed, fixed, and examined by confocal microscopy. (b) The quantitative results of Dil-labeled modified LDL binding to WT and $SE^{-/-}$ cells.

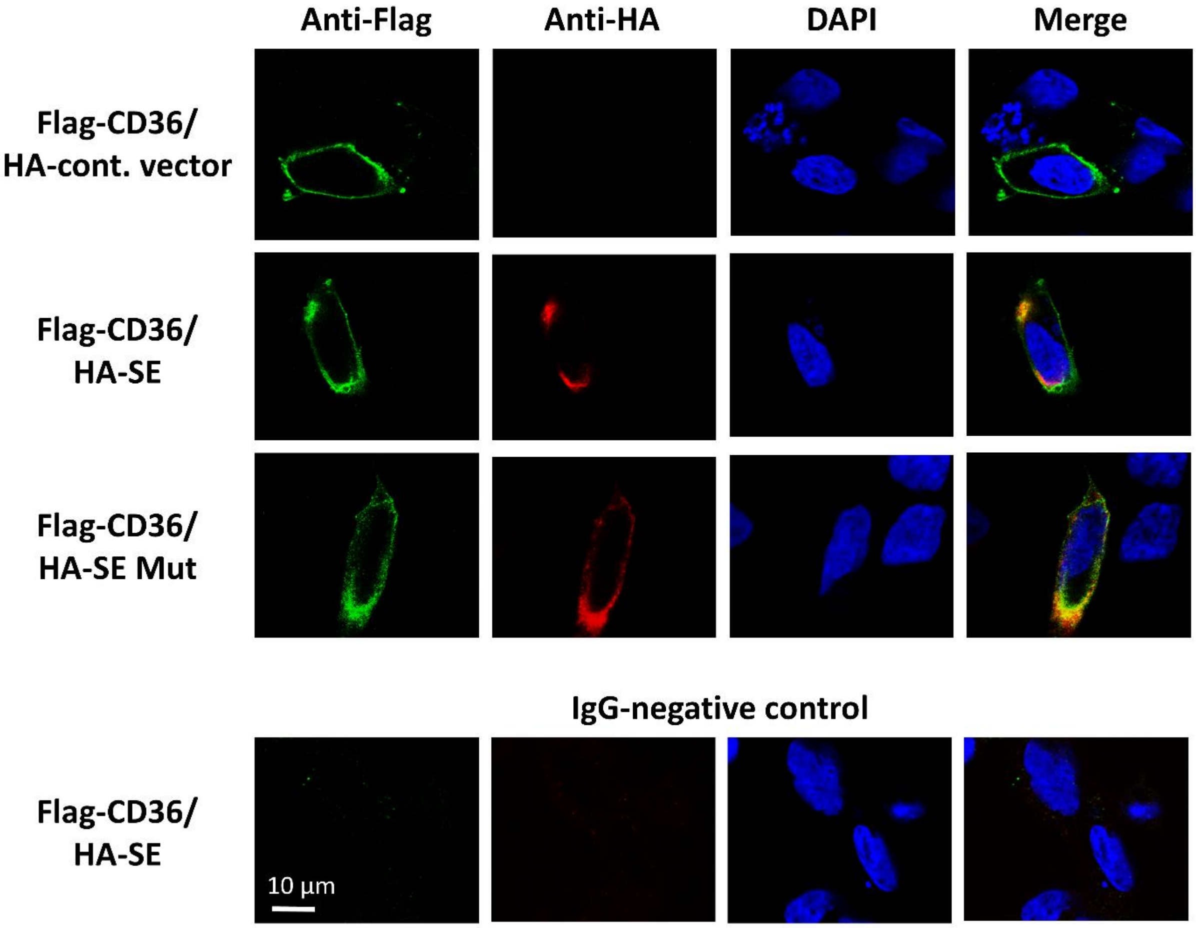

Figure S4. Surface coexpression of Flag-CD36 and HA-SE on transfected HEK293T cells. HEK293T cells transfected with FLAG-CD36 together with HA-empty control, HA-SE, or HA-SE mutant vector as indicated were fixed with 4% paraformaldehyde, permeabilized by 0.2% saponin, and blocked with 3% BSA and 5% goat serum in PBS at room temperature for 30 min. Cells were then incubated with mouse anti-FLAG M2 mAb and rabbit anti-HA- mAb, or control rabbit and mouse IgGs (negative control) at room temperature for 1 h. After 3 washes with PBS, cells were incubated with fluorescein isothiocyanate -conjugated goat-anti-mouse Ab and Alexa Fluor 568-conjugated goat-anti-rabbit Ab to detect FLAG- and HA- antigen-antibody complexes, respectively. After nuclear stain by 4′,6-diamidino-2-phenylindole (DAPI), cells were visualized by confocal microscope using 63x oil objective.

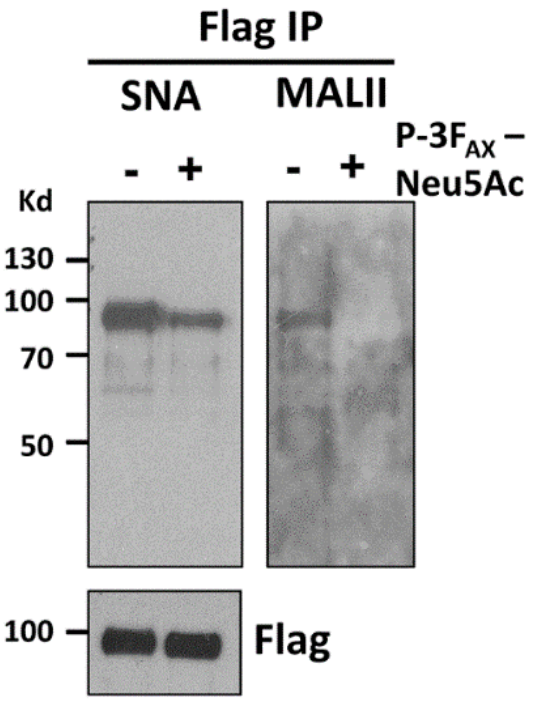

Figure S5. Analysis of sialylation status of CD36 overexpressed on HEK293T cells. HEK293T cells transiently transfected with Flag-CD36 vector were treated without or with 200 µM of P-3FAX-Neu5Ac for 66 h in culture. Cells were harvested and cleared cell lysates were subjected to immunoprecipitation with anti-Flag affinity resin. The imunoprecipitates were analyzed by lectin blotting with biotin-conjugated lectins as indicated or immunoblotting with anti-Flag antibody.

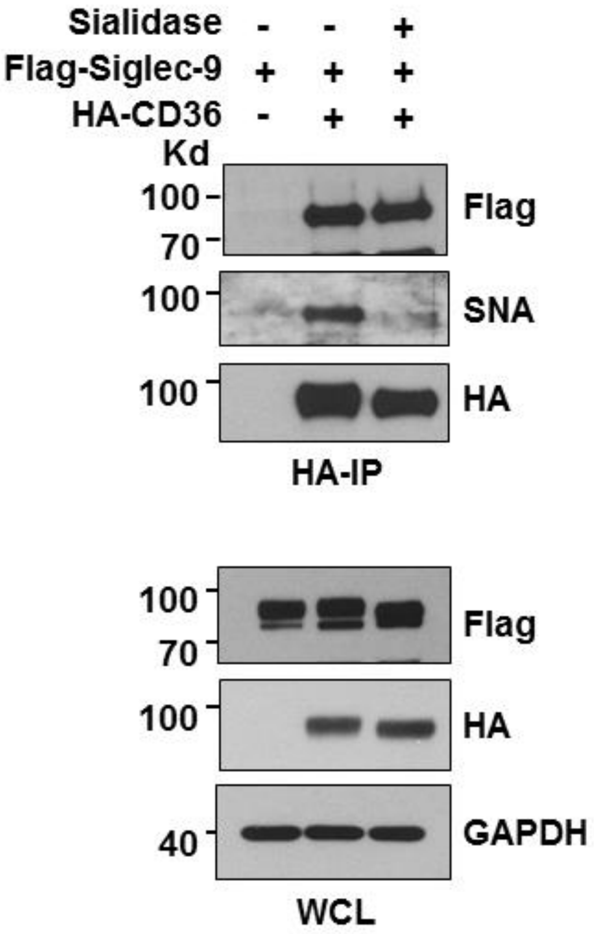

Figure S6. Siglec-9 interacts with CD36 independently of sialic acid. HEK293T cells were transfected with Flag-Siglec-9 plasmid together with or without HA-CD36 for 48 h. Cells were than treated with or without sialidase (0.1 unit/ml) at 37ºC for 30 min. Cell lysates were prepared and subjected to immunoprecipitation with anti-HA affinity resin. Immunoprecipitates were then subjected to Western blot analysis and lectin blotting with indicated antibodies and biotin-conjugated SNA, respectively.

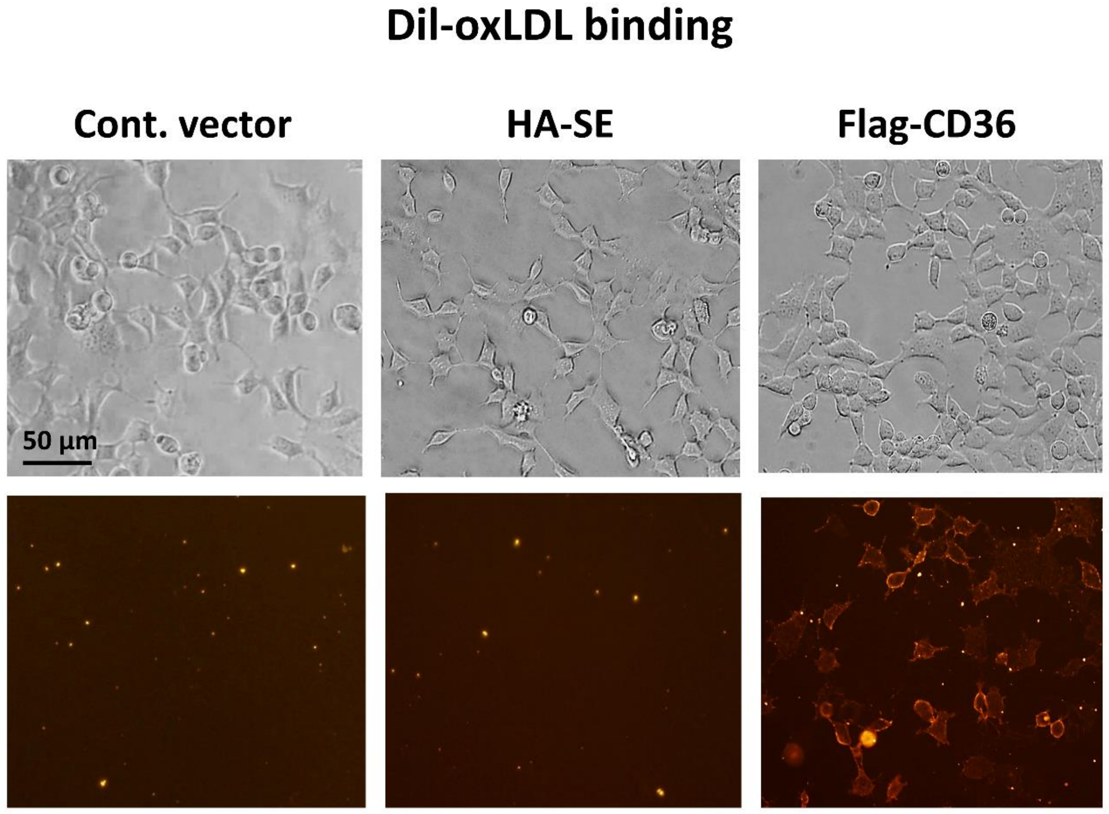

Figure S7. OxLDL binds to CD36 but not Siglec-E expressed on cell surface of HEK293T cells. HEK293T cells were transfected with control (empty), HA-SE or Flag-CD36 vector as indicated for 24 h. Cells were then incubated with 10 ug/ml Dil-oxLDL at 4°C for 1 h in culture. After three washes with HBSS, the binding of Dil-oxLDL on cell surface was examined by fluorescent microscope.

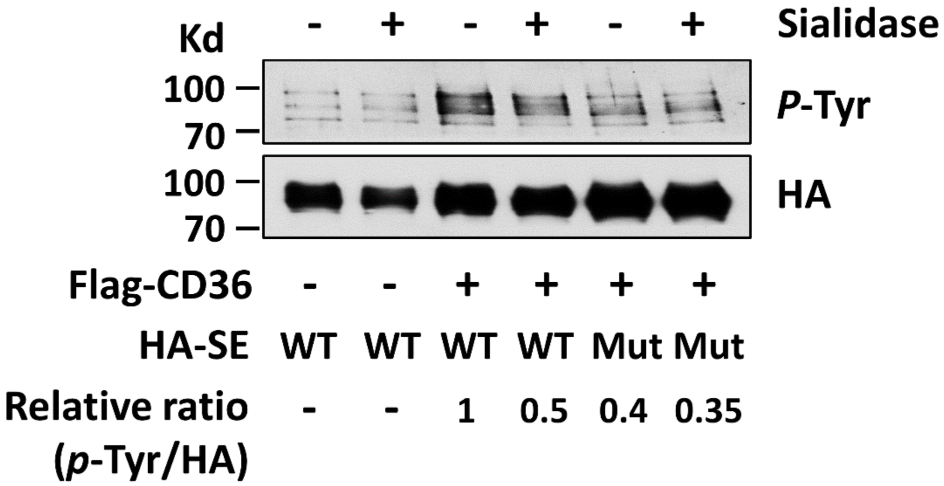

Figure S8. Sialidase pretreatment reduces oxLDL-induced tyrosine phosphorylation of WT HA-SE in cells coexpressing CD36. HEK293T cells were transfected with indicated vectors for 24 h. Cells were then incubated with or without sialidase (0.1unit/ml) at 37°C for 30 min, followed by treatment with oxLDL (50 µg/ml) in culture for another 30 min. Cell lysates were prepared and subjected to immunoprecipitation using anti-HA-tag resin. The immunoprecipitates were examined by Western blot analysis with indicated antibodies.
